# Virtual mortality and near-death experience after a prolonged exposure in a shared virtual reality may lead to positive life-attitude changes

**DOI:** 10.1371/journal.pone.0203358

**Published:** 2018-11-05

**Authors:** Itxaso Barberia, Ramon Oliva, Pierre Bourdin, Mel Slater

**Affiliations:** Event Lab, Department of Clinical Psychology and Psychobiology, University of Barcelona, Barcelona, Spain; Centro de Neurociencias de Cuba, CUBA

## Abstract

Mortality is an obvious if uncomfortable part of the human condition, yet it is impossible to study its impact on anyone who experiences it. Reports of phenomena associated with death such as out-of-the-body (OBE) and near death experiences (NDE) can only be studied post-hoc, since it is impossible to design a scientific study where an experimental group experiences death (and returns) and a control group does not. Yet NDEs seem to have a profound influence on the subsequent lives of people and are therefore worthy of study. Terror Management Theory, which argues that death anxiety contributes to in-group solidarity and hostility to out-groups, relies on studies that manipulate opinions and cannot be based on experiential evidence. Here we introduce a potential methodology that uses immersive virtual reality (VR) for the study of mortality and NDEs. Participants are embodied in alternate bodies in a beautiful island along with two companions. They explore the island and carry out tasks together. The mechanism of embodiment produces strong illusions of ownership over their life-sized virtual bodies. Over time each participant witnesses the death of the two companions and then her own death—which includes the reported features of an NDE (OBE, life review, the tunnel leading to white light) followed by a period of observation of the continuing activities in the virtual world on an external screen. Fifteen female participants experienced 6 sessions in the island, each starting as a child and gradually maturing, and eventually ageing and dying. Sixteen control subjects formed a waiting group. We introduce this as a methodology for the study of these issues, and present promising results, suggesting that those who experienced the island report life attitude changes, becoming more concerned with others and more interested in global rather than material issues compared to the control group. The results are based on a small sample size, and should be considered as indicative of the possibilities of this new methodology as a way forward for future studies in this field.

## Introduction

Understanding the experience and impact of death from a scientific point of view is problematic since the people who experience it are, of course, no longer around to report about what happened. The closest to this possibility are so-called Near Death Experiences (NDEs), where, typically due to coronary arrest, the patient dies, but is revived in a relatively short time period [1]. Such patients sometimes deliver compelling accounts of an event which includes an out-of-body experience (OBE) where they see what happened in, for example, the surgery room, but from an elevated viewpoint, they experience a life-review where their life flashes before them in various types of imagery, followed by a journey through a tunnel towards bright light, to emerge at the other end of the tunnel in a beautiful place, sometimes to be greeted there by deceased family or friends [2]. After revival patients may experience euphoria, a positive life renewal, where they become happier, more caring of others, and more concerned with universal issues rather than material ones [3–8]. This cannot be studied experimentally, since there can be no group that experienced an experimentally induced NDE with results compared to a control group that did not. Here we describe a study that shows how Virtual Reality (VR) can provide a methodology for the study of the effects of a (simulated) death on attitudes and behaviours of those who experienced it, compared to a waiting control group.

Apart from the reported effects of NDEs there has been significant research indicating that mortality salience influences the attitudes and behaviours of people [9]. The central theoretical construct is Terror Management Theory (TMT) which posits that the issue of our mortality is a critical and central question that may (non-consciously) shape adherence to core value systems and associated behaviours [10–12]. According to TMT people obfuscate the issue of death by identifying with global cultural norms that transcend the self. Hence in the presence of mortality salience people are more likely to emphasize their identification with societal norms, nationalism, pride in being member of a particular group, with the corollary of negating or despising individuals or groups that are considered as out-group from the standpoint of these norms. A further demonstration of this is that when presented with information that tends to affirm the possibility of post-death survival of the persona, such behaviour and attitudes are extinguished. ‘Symbolic immortality’ refers to the idea that although the self does not survive personally, there is survival, for example, through offspring, through influence that survives beyond any individual life, through one’s great works continuing on beyond death. Again evidence suggests that an increase in intensity of such symbolic immortality sentiments also extinguishes the specific effects predicted by TMT of negative reactions to out-groups [13].

A central methodological problem in the area of research discussed in this paper is that scientists cannot directly manipulate the independent variable ‘mortality’. People have no experiential knowledge of their own mortality, nor of post-mortality survival. There is no way to give one group, for example, an experience of post-death survival, and a control group not have this experience—and then observe the results with respect to their differences in attitudes or behaviour. Experiments in this field, therefore, typically use priming methods. In one study, for example, participants were given one of two articles to read—one that apparently provided scientific evidence for post-death survival of the persona based on studies of NDEs, or one that argued that NDEs were a phenomenal by-product of the dying brain. In other words, one group of subjects were led to believe in the likelihood of post-death survival and the other group not. When exposed to mortality salience the second group acted as predicted by TMT but the first group, having been given evidence of the possibility of survival, did not [14].

While these approaches are ingenious, they nevertheless operate at a highly abstract level. The manipulations involved necessarily remain at a conceptual level. There is no personal experiential aspect to them. Therefore, these experiments, that have provided highly interesting and informative results, are essentially experiments that show the relationships between ideas—how strengthening one idea might influence another idea, how the conjunction of two ideas produces a third idea.

Even the measured response variables themselves are abstract. For example, a typical approach is to elicit how much participants would punish a transgressor against societal norms, e.g., in the context of TMT experiments. But this relies on what people say they would do which is not necessarily the same as what they would actually do. Hence we argue that if people could actually experience ‘death’ and yet somehow survive this to live on in another form this would open up a quite different and exciting way to study these issues, and possibly lead to results that may have greater ecological validity, while maintaining internal validity, compared to previous studies in this area.

In this paper we show how to exploit shared immersive virtual reality (VR) to provide first person experience of a life cycle that simulates aspects of birth, childhood, maturity, decay, death, transition and post-death survival. An immersive, first person VR was created where groups of three participants, physically remote from one another, were simultaneously immersed for extended periods in a shared environment where they could see and interact with one another through gesture (but not voice). This VR was designed to be quite different from physical reality to emphasize its separateness, its ‘otherness’, existing on a different plane to our normal everyday reality. It was also designed to be a beautiful and interesting environment that people would not want to leave. Participants were ‘born’ into this VR embodied in humanoid but non-human virtual bodies. Participants experienced a period equivalent to childhood where they learned how to use their virtual bodies that appeared child-like, followed by a period of fully functional maturity with correspondingly mature bodies, then decay—where the body looked aged and frail. This took place over a period of 6 sessions over successive working days (apart from weekends). Each day each group of three participants carried out tasks together. Over time they saw each of their partners grow old and die in the virtual world, to be replaced by a new partner in the next session, initially represented as child-like. In each participant’s sixth session they experienced their own death, including a NDE, and they exited permanently from the environment. Once out of the environment they observed on a screen how the virtual world continued without them, in particular watching the two survivors build a memorial monument for their departed self. Overall the intention was to provide an experiential model of a complete life-cycle operating in a realm that is fundamentally different to our experiences in the normal world of everyday reality.

We designed this as a secular model to provide a possible and very simple explanation of one level of human existence: that we are living in a simulation, but unlike in the work of Bostrom [15], we ourselves are running our own simulation from some different plane of being to which we do not have access while in our earthly embodiment. At ‘death’ we go out of the ‘virtual reality’ that is our current life and return to live in whatever plane of existence we ‘really’ are. Of course, that does not solve the existential problem, since in that plane of existence, there may still be mortality. However, it is also a possibility that this other plane of existence is so utterly different to what we can imagine, that neither ‘mortality’ nor ‘immortality’ are relevant that ontology, being beyond the scope of our human consciousness to understand. We are not putting this forward as our view, but rather it is the idea implicit in the model that participants experienced.

VR is suitable for this research for two major reasons: first, it can lead to the illusion of participants being in a different place other than where they really are, and second it can lead to their illusion of having a different body. VR involves people entering and participating in a surrounding computer generated simulation of a world, experiencing the illusion of presence [16]. The critical element is that VR affords perception through natural sensorimotor contingencies where the whole body may be used for perception (e.g., through head gaze direction, through bending, reaching, touching) as in physical reality. We have argued elsewhere [17], and shown in many experiments—for example, [18–20]—that under these conditions, and when the VR is programmed to respond appropriately to the reactions of participants, then they tend to behave as if their experience were real.

In the experiment described in this paper we utilize a shared VR system which brings people who are physically remote together into the same consistent virtual world. They are each represented by a virtual humanoid body and they can see virtual representations of the other people present in the virtual world. Data communication is shared across a network, and consistency of the shared world is maintained, so that all involved perceive the same environment from their unique viewpoints, and can interact with one another.

Apart from presence, the illusion of being in the virtual world and the illusion that the events occurring there are really happening, there is also the illusion of body ownership towards a virtual body seen from first person perspective. Virtual embodiment refers to the substitution of a person’s real body by a life-sized virtual one spatially coincident with their real body. In the VR, through head-tracking, wherever the participant looks they would see the virtual environment. In particular, when they look down towards their own body the VR can be programmed so that they would see a virtual body that appears to replace their real one. Through real-time motion capture, when they move their real body, the virtual body can be programmed to move correspondingly in real time. Similarly, the participant can see reflections of their virtual body in reflecting surfaces such as mirrors. Seeing a life-sized virtual body from first person perspective substituting the real body, and the visuomotor synchrony that arises from motion capture data mapped to movements of the virtual body that correspond with real movements, typically give rise to the illusions of body ownership and agency [21, 22]. Body ownership is the perceptual illusion that the virtual body is the participant’s own body, and agency is the attribution of the actions of that body to the self. It has been shown that body ownership over virtual bodies of particular types can influence the attitudes and behaviours of participants. For example, both perception and self-identity are influenced when adults are embodied in the body of a child, and implicit racial bias by White people towards Black can be diminished through the embodiment of White people in a dark-skinned virtual body [23, 24] with this effect sustained over time [25].

**Fig 1:**
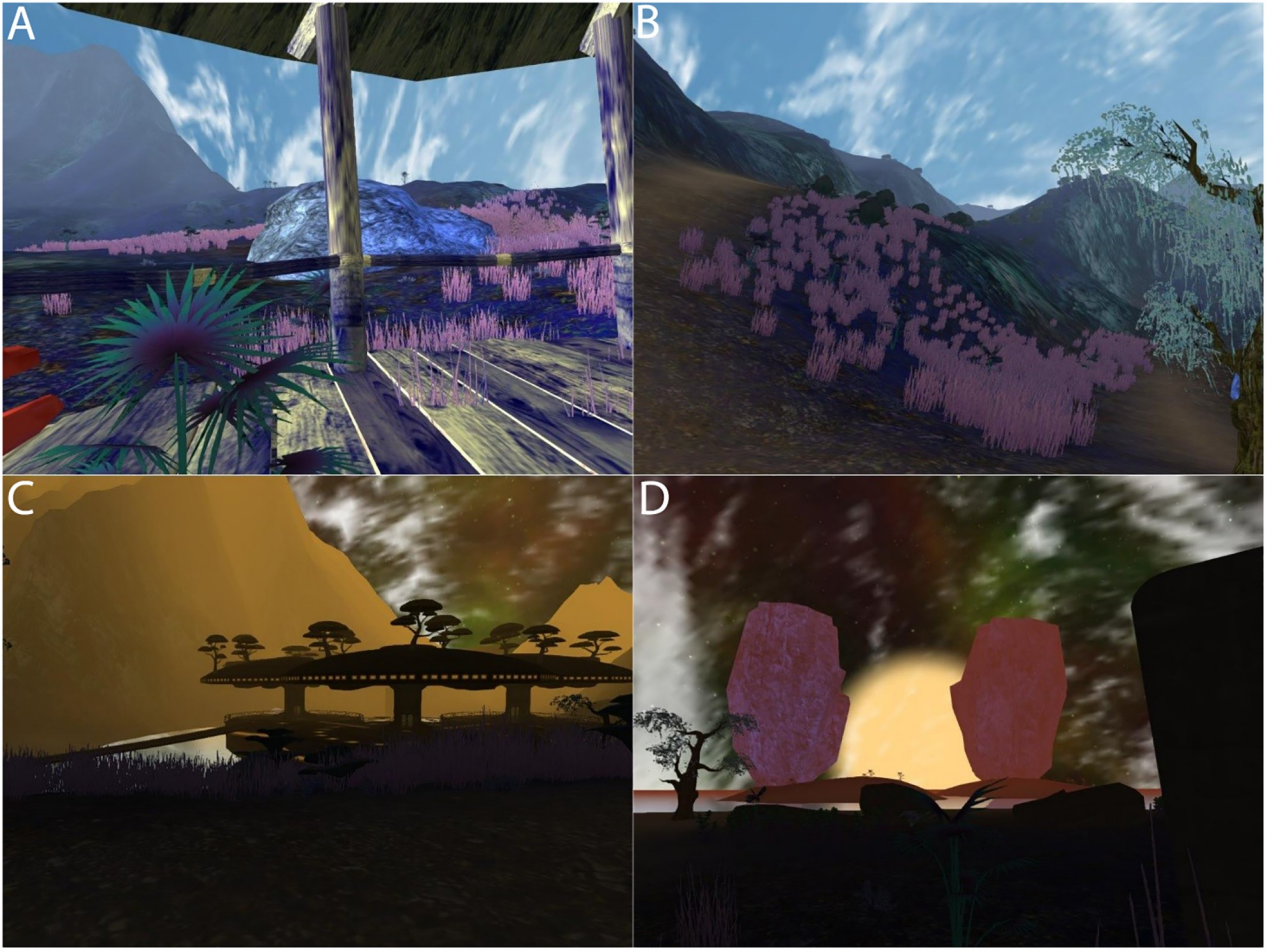
The Island environment. (A) A bridge with some mountains in the distance. (B) A mountain view. (C) Night falls. (D) The sun going down.

**Fig 2:**
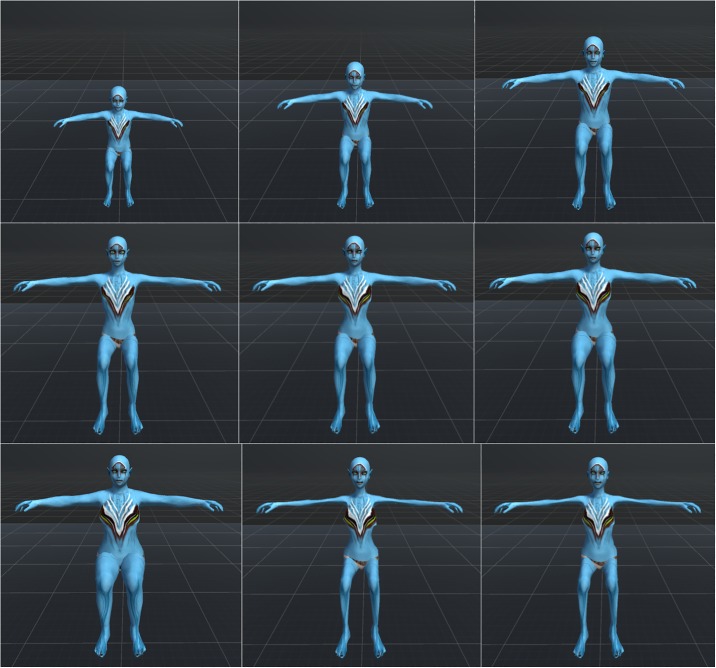
The virtual humanoid bodies and their evolution. Left to right, top to bottom—the body matures and then ages over time. This only shows the blue virtual body, the red and green are the same apart from colour.

Participants in our experiment were embodied in humanoid but non-human virtual bodies, located in a fantastic Island environment (Fig 1). The bodies were designed to be sex-neutral and generally appealing, reminiscent of the characters in the movie Avatar (http://www.avatarmovie.com). Their virtual bodies changed over time, first being child-like, then mature, and finally elderly (Fig 2).

A Video is available online (6:56 mins) that shows the major features of the experiment (youtu.be/C_eTXCqJObA). A 35-min video documentary is also available (vimeo.com/128820422).

Our main goal in constructing this VR simulation was to provide a methodological foundation in which mortality/immortality can be used as an independent variable in research. As well as introducing this methodology our secondary goal was to explore how the experience impacted the subsequent attitudes of the participants in relation to three outcomes. The first is concerned with their death anxiety—how would their encounter with simulated death, both of their partners and their own post-death survival, impact their death anxiety? Second, given their encounter with death, how far would the predictions of TMT hold up—in particular would they tend to demonstrate a diminished defence of their nation? Third, and the most important, since they had experienced a NDE would they report changed attitudes to life—supporting more global rather than mundane concerns, as is often reported for people who had experienced an NDE through, for example, cardiac arrest as discussed in the opening paragraphs?

The major hypothesis of this research is that a simulated NDE and the implicit model of survival beyond death would result in evidence of life-change as measured by the Life Changes Inventory [26], compared to a control group that did not have this experience. Second, we were interested in the predictions of TMT. Experiencing the implicit survival beyond death should according to TMT result in an impact on the world view of participants to be less defensive of symbolic survival categories such as defence of their nation. Third, following on from our own previous work, where we found evidence that a simulated OBE results in a reduction of death anxiety [27], we were interested in whether those findings would be reproduced in a more complex scenario, where participants witnessed the simulated deaths of others, as well as experiencing their own NDE, and an implicit model of survival beyond death.

## Methods

### Experimental design and recruitment

We used a single binary factor between-groups experimental design. The Control group (n = 16) was a waiting list group, and the Experimental group (n = 16) experienced the VR. The groups consisted only of female participants, with mean and S.D. age 20.0 ± 1.50 (Experimental) and 20.3 ± 1.40 (Control). Participants were comparable across a range of demographic indicators (see S1 Text).

Two advertisements addressed to Psychology undergraduate students were posted in the webpage of the Department of Basic Psychology of the University of Barcelona, one referring to a 3 sessions experiment (for the Control group) and the other referring to a 7 sessions experiment for the (Experimental group). For both groups the first meeting was a screening session after which only the participants who matched with the appropriate profile for the experiment would be given the option to continue. The advertisement requested participation from adult females with Catalan as their mother tongue, who were not taking any psychoactive medication and, in the case of the experimental participants, not suffering from epilepsy.

During the screening session, applicants had first to complete questionnaires that checked that they were not affected by any recent death in their family or close friends, and to assure that they had a feeling of Catalan identity. This was relevant for our TMT measure, which was also the reason for asking participants having Catalan as their mother tongue. We required that all recruited participants should score at least 20 out of 40 on the National Identity questionnaire that we adapted from [28]. The screening also included the LSB-50 questionnaire [29, 30] that assesses psychological symptoms. Taking as a reference the scores of the general population, applicants with 97th percentile or more on the IRPSI (*Índice de Riesgo Psicopatológico*) subscale, or with the severity global index equal or superior to 97th percentile, or with 2 subscales equal or superior to 97th percentile were screened out. Additionally, in the same screening session participants were asked to complete a demographic questionnaire and a self-esteem scale [31, 32] (translation by [33]), but these were not used as an exclusion criteria.

Finally, during the screening session all recruited Experimental group participants had a short Virtual Reality experience in order to ensure that they could experience body ownership over a life-sized female virtual body in which they were embodied from first person perspective. They answered questions on a Likert scale from 1 (not at all) to 7 (totally) regarding their illusion of body ownership, and the minimum required score for inclusion was 4. The questions were: ‘Though the body I saw in the mirror did not look like me, I had the feeling it was my body’ and ‘Although the body I saw when I looked down did not look like me, I had the feeling it was my body’. A third question checked that participants did not feel dizzy while in the virtual environment and the maximum accepted level of dizziness was 3 (out of the maximum of 7 meaning the greatest feeling of dizziness).

Among all applicants, in total, 14 were screened out (5 for attendance incompatibilities, 3 for LSB-50 high score, 2 for recent death in their family or close friends, 1 for not satisfying the Catalan criterion, 1 for her previous participation in an incompatible study and 1 because she did not reply after the screening session). Finally the 32 participants complying with the requisites of the experiment were recruited. However, during the course of the experiment one participant was invited to end her participation due to her discomfort due to simulator sickness during virtual reality sessions. This reduced the experimental group to 15.

Participants in the Experimental group were paid a total of €120. They were paid €5 for the initial screening session (S0), then for the VR sessions €5 for each of the first two sessions (S1 and S2), €10 for S3, €15 for S4, €20 for S5 and €30 for each of S6 and S7. The final debriefing session (S8), to which all Experimental participants were invited to discuss their experiences together in a structured way, led to an additional payment of €15.

The control group were paid €35 in total for 3 sessions (€5 for the screening session, €10 for the first session and €20 for the last session).

The experiment was approved by Comissió Bioètica of Universitat de Barcelona. All participants gave their written informed consent prior to participating to the experiment on a form devised for this purpose that had been approved by the said Comisió de Bioética. The study was performed according to institutional ethics and national standards for the protection of human participants.

### Materials

The application was executed on 4 PCs and was implemented in Unity3D (Unity Technologies, Copenhagen, Denmark). The 4 PCs were connected via a dedicated 1 GHz LAN network, with of the PCs acting as clients and the remaining one as a dedicated server. The 3 client PCs were each assigned to a participant. Each also had a Kinect 2 sensor (Microsoft Inc., Redmond, Washington, United States) and a head-tracked stereo head-mounted-display (see below). The Kinect 2 sensor was used to capture the upper body movements of seated participants and for gesture recognition. The upper body real-time motion capture was achieved with the Kinect depth sensor, allowing marker-less full-body motion capture with advanced skeletal tracking [34]. The position and orientation of the various bones defined by the skeleton were applied to the participant’s virtual body bones by our custom software. The gesture recognition system was initially based on the Gesture Recognition Toolkit [35] but finally it proved more efficient to write our own program for this.

The VR application was displayed in Oculus DK2 head-mounted-displays (HMD; Oculus VR, Irvine, California, United States). The Oculus has a 1080p OLED panel, split to 75 Hz 960×1080 per eye rendering. The field-of-view is approximately 106° vertical by 95° horizontal. The Oculus internal head-tracking was used to obtain head rotation and orientation and the Kinect was used for head position tracking, thus resulting in 6 degrees of freedom head tracking.

The virtual environment was created starting from the demonstration ‘tropical paradise’ released in 2007 with ‘Unity3D 2.x’ by Unity Technologies. It was upgraded to be compatible the later version of Unity3D v4.6.9f1, and then heavily modified (terrain, textures, shaders, vegetation) and optimised for rendering in VR. Some additional 3D models also originating from example demonstrations and tutorials by Unity Technologies were incorporated to the project to produce the resulting alien Island (Fig 1).

The virtual bodies were first created using the DAZ3D (http://www.daz3d.com) system based on their available models. Then they were exported to Maya (http://www.autodesk.com/maya) to be fine-tuned and optimized. They contained blend-shapes that allow dynamic changes to their appearance. Our custom software included C# scripts to control the evolution of the body in order to produce smooth transitions from the child to the elderly version over the six sessions.

### Organisation of the VR sessions

Each participant in the Experimental group had one VR session per day for 6 consecutive working days, always at the same time of the day and in the same room. Each session included three participants (although in the early sessions one or two confederates were used before enough actual participants had been ‘born’ into the virtual world, explained in more detail below). An experimenter was assigned to each participant. Participants were physically located in separate adjoining rooms and great care was taken to ensure they never met or even saw each other during the experiment—for example, they entered their room at different times to avoid meeting. Participants were never told anything about the other virtual characters (i.e. that they were other participants, or experimenters, nor that they were in adjoining rooms).

**Fig 3:**
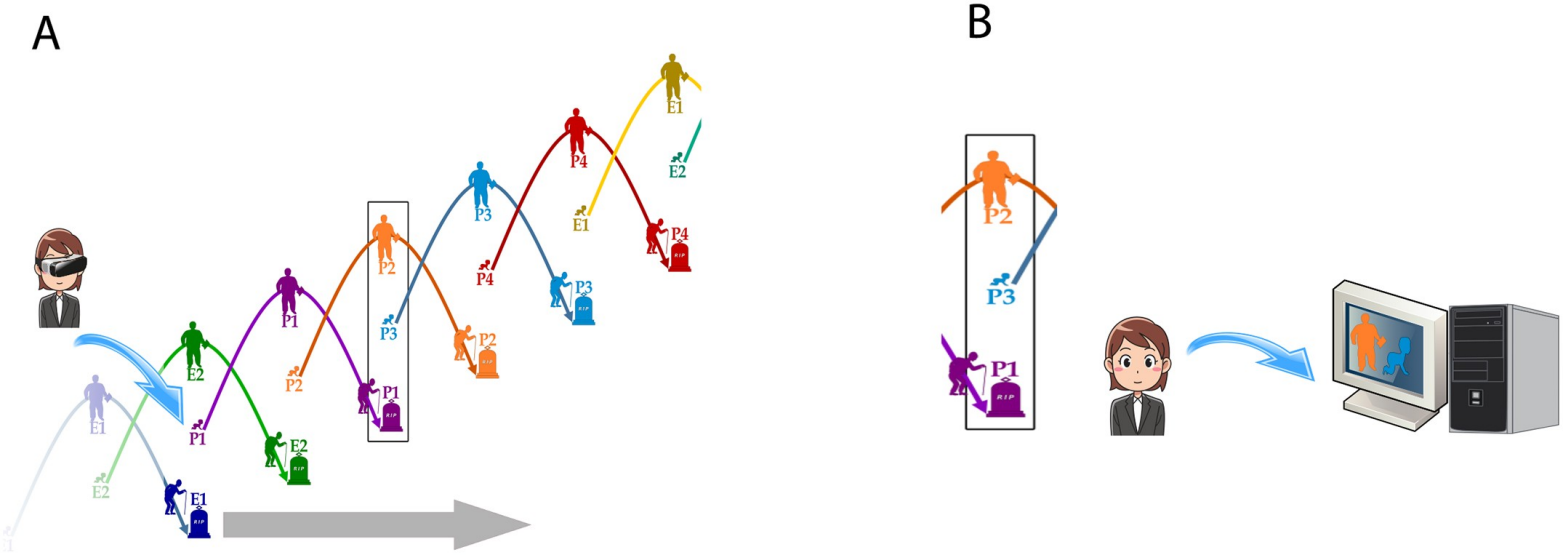
Organisation of the experiment. (A) Shows that at the start there were two confederates (experimenters) E1 and E2 and one participant (P1). When E1 ‘died’ participant P2 joined, and so on. (B) When P1 eventually ‘died’ she observed the scenario on a computer screen outside of the VR.

Fig 3 illustrates the arrangement whereby participants joined and left the study. It was important that the various activities to be carried out by the participants during their VR exposures should be figured out by themselves, rather than receiving instructions from the outside. This was also designed to foster collaboration between them. Hence when the first participant (P1) entered the environment the other two characters (E1 and E2) were confederates. This is shown in Fig 3A. Eventually one of the confederates (E1) became old and died, to be replaced by a participant (P2). At this stage therefore, there were two participants (P1 and P2) and one confederate (E2). Eventually E2 died, to be replaced by P3, leaving 3 participants—P1, now old, P2 mature, and P3 a child. This continued as participants died and were replaced. Fig 3B shows that after a participant died, for the remainder of that session there were two remaining participants, while the ‘deceased’ one continued to observe the rest of the session on a screen from outside. This process continued across all participants, and similarly for the last participants, confederates had to join in order to always keep the number in the virtual world as three for the start of every session.

The Control group was a waiting group where after all the Experimental group participants had completed all of their 6 sessions, the members of the Control group completed the same questionnaires and tests in parallel with them. Each Experimental and Control group participant completed all of their procedures without having any contact whatsoever with other participants.

### Embodiment and interaction

The participants were seated throughout. They were instructed that they could move only the upper part of their body (torso, arms and head) but not their legs. They would see a life-sized virtual body spatially coincident with their own body from first person perspective (1PP), so that when they looked down towards themselves they would see the virtual body substituting their real one. The Kinect was used to track their body movements in real time, and the corresponding movements were mapped to the movements of their virtual head and upper body and limbs. At various times participants would see their body in a reflective surface, in addition to seeing it normally when they looked towards themselves. Hence embodiment was executed through 1PP and visuomotor synchrony between their real movements and movements of the virtual body.

The virtual bodies were humanoid but not human. Their sex was not clearly defined being rather androgynous. The body of each participant was coloured red, green or blue, and remained the same throughout their entire life-cycle, with no two the same colour. During their six VR sessions, the virtual body of each individual evolved, starting as young child at the beginning of the first session and ending as a frail old character that died during the last (6^th^) session. Fig 2 depicts the evolution of the virtual body over the life-cycle.

Participants would see that they were seated on top of cloud-like platforms, and that their two partners were also seated on such a platform. These platforms also acted as their vehicle for movement through the virtual world. Participants had learned that they could move through the world by leaning in the direction of the desired location. The leaning angle of the upper-body, as measured by the Kinect placed in front of the participant, thus moved them in the direction of the lean. This system measured the leaning forward angle of the upper body to control the speed of forward movements and the backwards angle to move backwards. Left or right rotations, were based on the measured lateral leaning angles. To minimise the chance that participants would become sick when moving, a very slow linear and angular speed was used, as well as the linear and angular acceleration. This moving metaphor, based on an original idea by Fairchild, Lee, et al. [36], was sufficiently intuitive and effective to allow all participants to move inside the virtual environment even though they had quite short training (see below).

**Fig 4:**
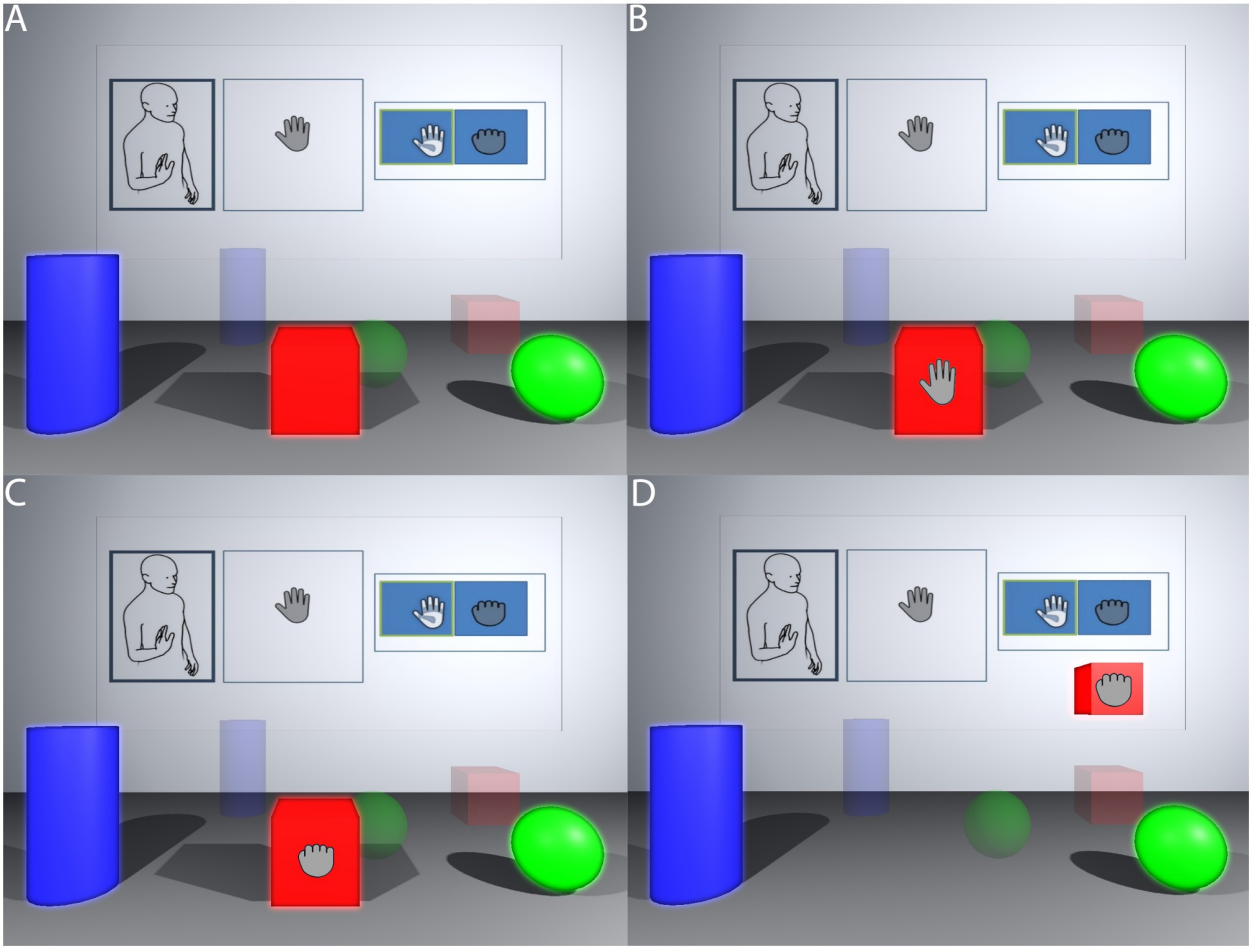
Interaction with Objects: The training room. (A) Three objects are shown and the hand cursor. (B) The participant moves their arm and hand to place it over an object. (C) She closes her hand to grab the object. (D) The object then moves with the participant until the hand is opened.

To be able to interact with the objects of the virtual world, participant had to first bend their right elbow at 90°, the palm facing the Kinect, as shown in Fig 4. Once this gesture was recognized, an open hand virtual cursor appeared on participant’s view. She could move this cursor by moving her real hand to point at objects of the same colour as her body (red, green or blue) and could only interact with such objects. Once the cursor was above a moveable object, it started blinking showing an open hand cursor followed by a closed hand cursor repeatedly (Fig 4C). At this moment if the participant closed her hand, she would grab the virtual object. When the object was grabbed, it was linked to the participant, so that if she moved through space, the object would move with her. To release the object the participant had to open her hand.

There was no sound communication between the participants, but they used gestures to communicate: e.g., pointing and waving to one another.

### Events and the scenario

When a participant entered the environment for the first time (at the beginning of the first session), a 2 minute voice tutorial was automatically played teaching her how to navigate the virtual world, i.e., introducing the leaning metaphor. The tutorial took place in a virtual room decorated with large mirrors, so that participants could see their appearance.

Once this first tutorial was finished, the participant was automatically teleported to a second tutorial, where she was taught how to interact with objects of the virtual world. As mentioned earlier, each participant had one of the three colours (red, green or blue) and they could interact only with the virtual objects of the same colour. Their main task during the tutorial, was to move some objects from one place to another specific place, by pointing at them, grabbing them, and moving them to the target location. This second tutorial also lasted 2.5 minutes.

After this second tutorial the new participant was familiar with the main skills necessary to interact in the virtual world, and was automatically teleported to the Island, in a fenced area where she had to wait for the two other participants to join. The other two participants were both in their later sessions and instead of these tutorials, they carried out some exercises in a virtual room (‘embodiment room’), where they were asked to move the upper part of their virtual body in front of a virtual mirror. The purpose of this embodiment training was to familiarise participants with their virtual body, and also to allow a period for development of the illusion of body ownership over that body. Recall that participants in their very first session also received this feedback through seeing their reflected virtual body in the mirrors in the navigation training session. After 1.5 minutes of exercises, the participant was automatically teleported into the Island, to a fenced area where she had to wait for the others to join before being able to continue.

The Island covered a large area with colourful alien vegetation and mountains (Fig 1). To give participants the sensation of the time passing more than the real elapsed time, we implemented a day-night cycle (Fig 1). Apart from increasing the realism of the virtual world, the purpose was to induce in the participants the idea that the time was passing to match the evolution of their ageing virtual body. The day-night cycle time was adjusted to last a few minutes, so the participants could see the sunrise and sunset several times per session. Finally, the landscape (Fig 1) of the Island was designed to be singular and unusual, but also visually attractive so that the participants would appreciate their stay in the virtual world.

The session on the virtual Island lasted for about 13.5 minutes. In order to ensure that all participants spent the same time in the virtual Island, when there was a session with a newcomer, the new participant entered first into the VR, and while she was doing the second tutorial about interacting with objects, the other two participants entered into the VR and did the corresponding embodiment stage. This was because tutorials required more time (5 minutes in total) than the embodiment stage (1.5 minutes). If all the participants were in a later stage (S2-S6), they simply entered in VR at the same time and all experienced (separately) their corresponding embodiment stage. So, in both situations, we ensured that all the participants arrived at the fenced area approximately at the same time, and that they spent the same time in the Island.

To summarise: prior to their first session participants would receive (i) navigation training (ii) object selection and placement training. In subsequent sessions they would experience (iii) the embodiment training only. Immediately after (i) and (ii), or (iii) they would be transported to the fenced area of the Island. The fenced area was used as the arrival point in all sessions, and served as a waiting room and also gave time for all participants to be connected to the server. This way we could synchronize all the participants, in spite of the varying times they needed to put on the HMD and go through the trainings.

**Fig 5:**
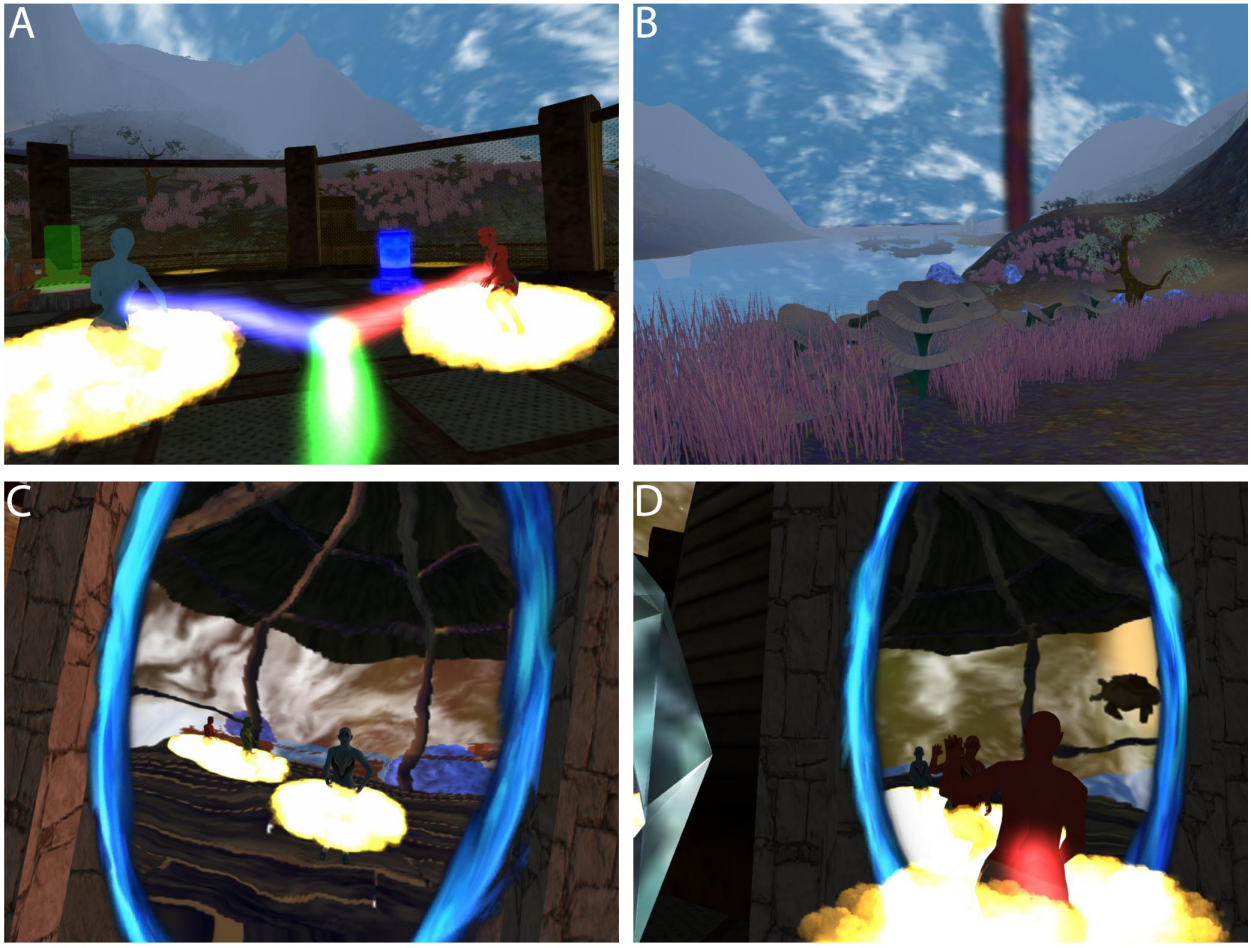
The shared virtual environment. (A) Three participants simultaneously embodied—from the viewpoint of the green character. The cord links the three together. (B) The column of smoke indicating the location of the available task. (C) The mirror portal. (D) The ‘red’ participant approaches closely to the mirror portal.

From the moment they entered the Island, participants were connected to each other by an illuminated rope (or “cord”), as shown in Fig 5A. This encouraged them to be close to one another, since if they separated more than a specified distance from the centre of the triangle formed by the links between them, an alarm would sound, and they would not be able to move further from this centre. This was to increase the likelihood that the participants would have the feeling of belonging to the same group and to enhance their cooperation in carrying out the tasks they had to perform.

Once all three participants joined the fenced area, a mirror portal was activated (Fig 5C and 5D). This portal had a double function, first they would see the reflection of their virtual body in the mirror, which would be likely to reinforce body ownership and ensure that they knew their own appearance. Second, by crossing through the mirror portal they would teleport to another part of the Island. Participants could see an overview of the other side of the portal in the mirror. At each session, when crossing the portal participants were teleported to a location close to where the collaborative task available that day would take place.

The location of the task was indicated by a large purple column of smoke (Fig 5B). This column disappeared when participants were close enough to the location of the task. However, after crossing the portal participants were free to explore the Island as they wished, apart from the cord that united them. They had no other obligation than stay together as determined by the cord. Nevertheless, participants tended to go to the task locations and collaborate to achieve the tasks proposed, typically led to the task location by the ‘older’ members of the group—recalling that newcomers were always with two more experienced participants (or confederates in the early sessions).

Every day in sessions S1-S5, after 13.5 minutes from when all participants had been connected to the server and joined the fenced area of the Island, the environment faded to black and participants were automatically teleported to the embodiment room, where they had to perform the embodiment exercises in front of the virtual mirrors. Again, the goal was to show the participants the current state of their virtual body—in effect how they had aged during the session.

### The collaborative tasks

To keep the participants occupied and entertained, and to reinforce the social bond between them, we designed several collaborative tasks that were available in every session. These tasks were designed to require cooperation amongst the three participants to be completed. There was no reward for completing the tasks and no obligation to do them—participants could choose to spend their time exploring the virtual world instead of doing the available tasks. Recall that each participant was consistently (from S1 to S6) embodied a virtual character that could be either red, green or blue, and in those tasks that required interacting with objects each participant could interact only with the objects that matched their own colour.

A new task was available for each session, each time at a different location. There were three main types of tasks, with two variations each, which formed six different tasks:

- *Classification task*: In this task, there were two baskets and some objects nearby. The participants had to put the objects in their corresponding basket. If an object was placed in the wrong basket, it was rejected and thrown out.
- *Maze task*: participants had to exit from a labyrinth by opening some doors which blocked their way. To deactivate those doors, each participant had to remove her key (represented as a coloured cube) from the door. Once all the locks were removed, the barrier disappeared and participants could continue to follow their path looking for the exit of the maze.
- *Piano task*: for this task participants had to pass over the lit key of a huge piano keyboard that appeared on the ground. Only one key was lit at a time with a red, green or blue colour, and when the corresponding participant (i.e., the red, green or blue one) passed over the key, the key was turned off and a small piece of music was played. Then another key of another colour was enlightened, until the three participants had passed over their key, thus playing a short tune.

### Virtual death

The sixth and last VR session for each participant began as the others with the exercises in front of the mirrors in the ‘embodiment room’. By this time their virtual body was old and frail (Fig 2). Then they were transported to the Island as previously but after approximately 7 minutes the experimenter supervising the participant initiated the virtual death process (by a key press on the controlling computer).

**Fig 6:**
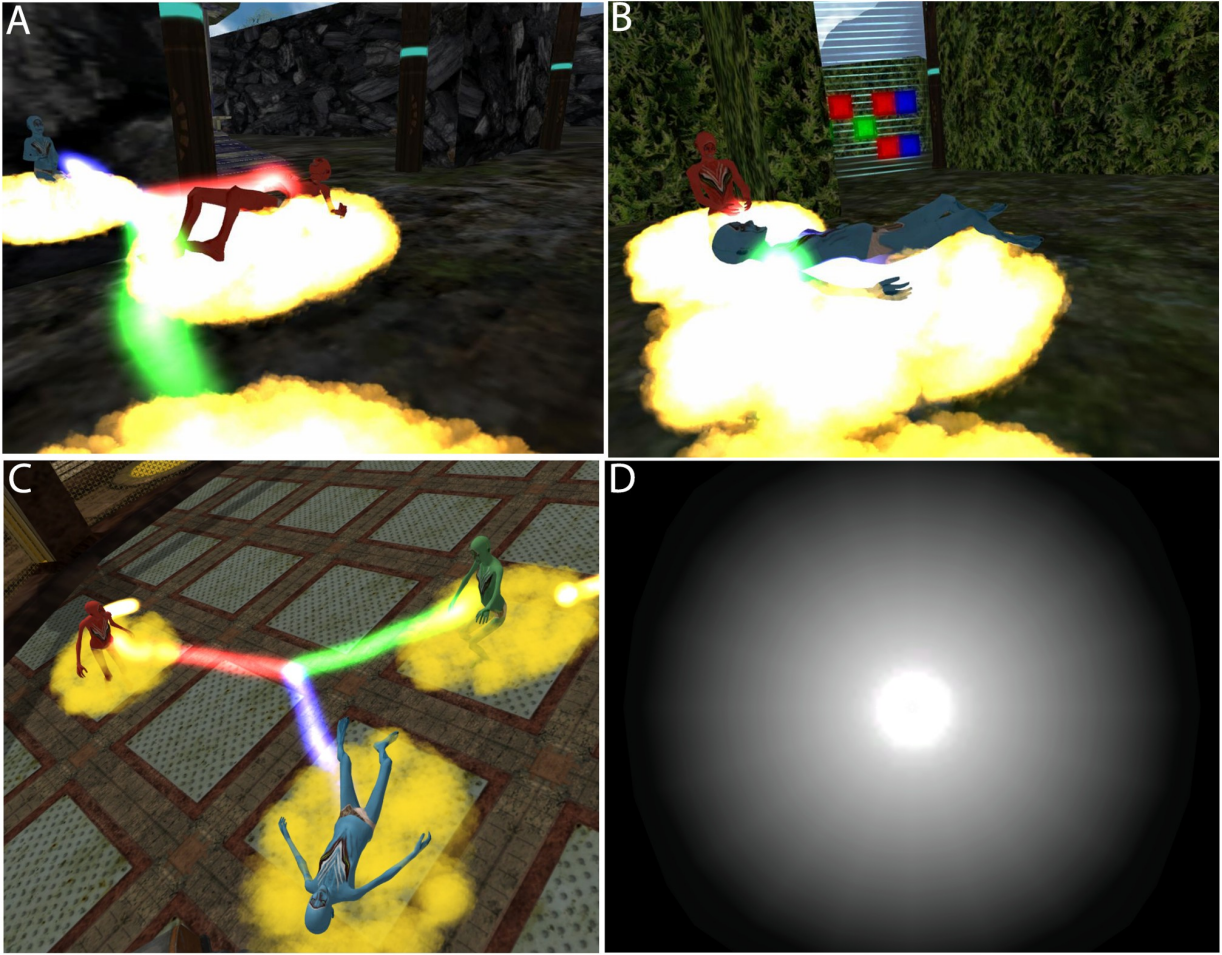
The virtual death. (A) Other participants see the deceased character lying on its cloud. (B) They typically approach the dead character. (C) View from the out-of-body experience of the dead character. (D) The white light at the end of the tunnel.

For the participant the virtual death process started by her vision becoming blurred through three flashes, then she could hear the beats of her virtual heart and after some seconds there was a cry of pain. Further, her viewpoint was slowly moved above her now immobile virtual body lying on the cloud, as shown in Fig 6B, to generate an Out of Body Experience (OBE) [27]. Then, the disembodied participant was teleported to a dark room where she would see a life review of her virtual life. This was through a rapid playback of a succession of images recorded during the different sessions of the participant’s virtual experience, presenting a summary of her life in images based on the idea of the life-review flashbacks reported by some people who have experienced a NDE [37]. Once the life-review was over, the participant would see a distant light at the end of a dark tunnel and she was automatically attracted towards it, giving her the sensation she was moving through the tunnel until reaching the light. At that moment everything faded to intense white as also reported in NDEs (Fig 6D).

After that, the experimenter removed the HMD from the head of the participant, and invited her to sit in front of a computer screen where she could see, now from the outside, events continuing in the virtual world.

**Fig 7:**
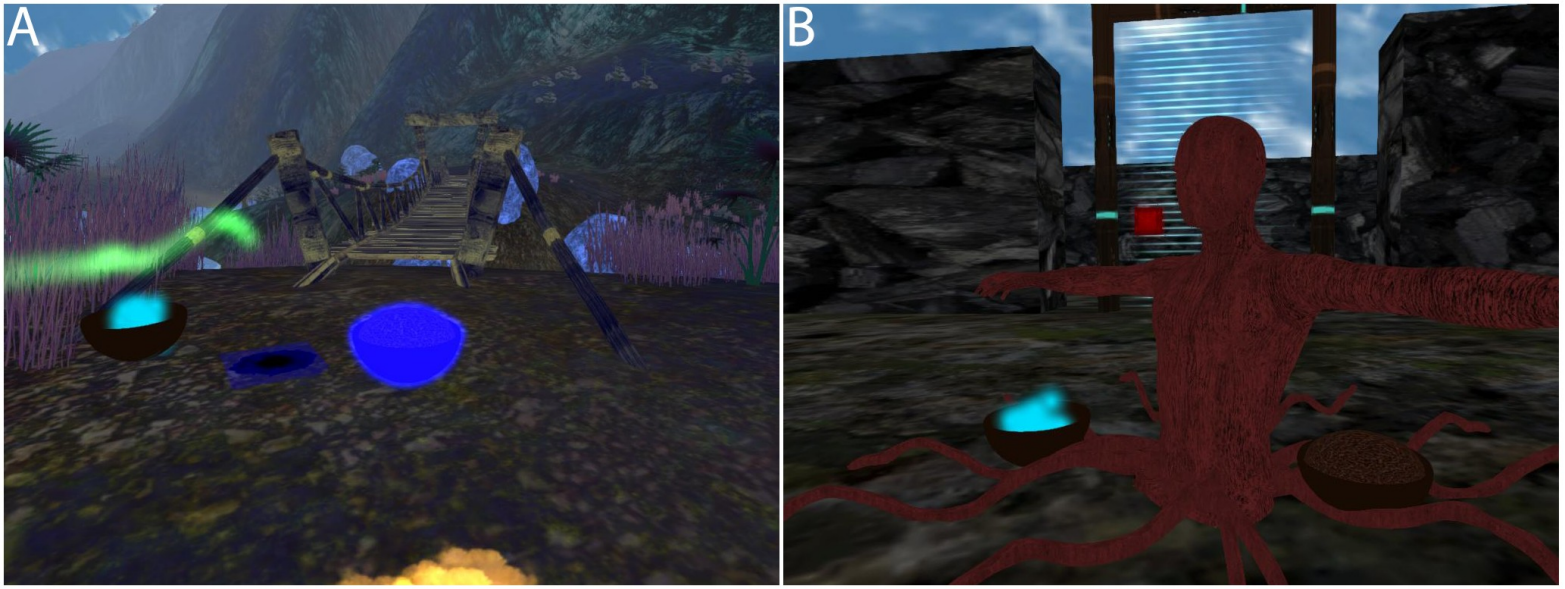
The ceremony. (A) The bowls used in initiating the ceremony. (B) A tree grows but in the form of the deceased character.

Meanwhile, in the virtual world, the remaining participants typically approached the dead body lying on its cloud (Fig 6B). While the participant was seated in front of the screen, the experimenter discretely initiated a ceremonial task. The ceremony started with the disappearance of the dead body and the cloud that was supporting it to be replaced nearby by two bowls filled with seeds of the two colours of the remaining participants. One of the bowls was lit. The participant with the same colour as this lit bowl had to grab it and move it over to another bowl containing a blue luminescent liquid that had appeared at the former location of the virtual body (Fig 7A). After the participant completed this ceremony, the bowl of her colour disappeared and other bowl, that of the remaining participant, was lit. Once the second participant had grabbed her bowl and moved it over the bowl containing the blueish liquid the roots of a tree slowly started to grow out of the ground. However, rather than a regular tree, what eventually grew was a statue of the same colour and appearance as the deceased character (Fig 7B). The final size of the statue was approximately 1.5 times the size of the virtual body. The participant who had died in the virtual world observed this ceremony and monument to her former self on the computer screen. The experimenter could also force the wooden statue to appear if the participants were unable to accomplish the ceremony.

### Data collection

Daily, after their VR session, each Experimental participant completed a post-trial questionnaire on presence, evaluation, body ownership and agency (see next section) and was interviewed. Participants were also asked to keep a daily diary at home, in which they were asked to express any thoughts and feelings they had in relation to the study. Moreover, on day 6 (the day of their virtual death), after the post-trial questionnaire and before the interview, they were exposed to a mortality salience manipulation (as explained in the next section). Finally, they were asked to attend the day after their last VR session (S7) to complete two questionnaires on fear of death, a Life-Change inventory and an Implicit Association Test (see next section for the description of all these measures).

Participants in the Control group were each matched with one experimental participant, in the sense that they completed the questionnaires that each experimental participant had completed on days 6 and 7 at the same time. Control participants did not have any VR experience and only came for two days, coincident with the days in which each experimental participant’s session 6 and 7. Therefore, controls were exposed to the mortality salience manipulation on their first day and completed the fear of death questionnaires, the Life Change Inventory and the Implicit Association Test on their second day.

The Experimental participants never met each other throughout the whole period of their involvement. However, they were invited to attend a final day (S8), where they all could meet and have a group debate and extended debriefing. They were told in advance that they would meet the other participants involved in the experiment so they could decide if they wanted to participate in this final session or remain anonymous.

Analysis of the diaries, post-experience interviews, and the results of the final meeting are not included in this paper, and is the subject of further work.

### Response variables

There were several categories of response variable: those providing background information, presence and body ownership, death anxiety, Terror Management Theory and a life changes inventory.

**Background information** was provided on several variables in questionnaires administered prior to the first VR session for those in the Experimental group, and during the same period for those in the Control group. There were a number of demographic variables such as age, occupation, extent of computer game playing, religious belief, which are summarised in S1 Text. Self-esteem (*selfesteem*) was assessed with the Spanish version [33] of the Rosenberg Self Esteem scale [32]. This consists of 10 questions each on a 1–4 scale (from *totally disagree* to *totally agree*) where the higher values indicate greater self-esteem. The overall score is the sum of these 10, so that the scores range from 10 to 40.

**A post-trial questionnaire** was administered at the end of each of the 6 sessions to assess presence, participants’ evaluations of the experience, and body ownership and agency. Presence was assessed using a variant of a questionnaire that has been used many times before—see e.g. [38]. These are the questions *there*, *real*, *happening*, and *copresence* in Table 1. Evaluation of the experience was concerned with participants’ responses to the others with whom they shared the world, comfort, and the task (*friendly*, *collaborate*, *uncomfortable*, *likedtask*). Body ownership was assessed using questions that have been used before—e.g., [39] and are shown as the questions *mirror*, *down*, *mybody*. The question *mymovements* addresses agency, whether the participants attributed the movements of their virtual body to themselves. This is mainly a test of the system, since it was designed to give synchronous visual-motor feedback for the upper body.

**Table 1:** The post-trial questionnaire on presence, evaluation, body ownership and agency. Each question was in the form of a statement. This was scored on a 7 point Likert scale where 1 indicated that they did not feel that sensation at all, and 7 that they felt that sensation in each maximum intensity.

Variable name	Question
**Presence**	
*there*	I had the sensation of being in the virtual world.
*real*	There were moments when the virtual world seemed more real to me than the laboratory in which the events were really taking place.
*happening*	I had the sensation that the events taking place in the virtual world were really happening.
*copresence*	I had the sensation to be sharing the virtual world with the other two beings, as if I really were with them in the same place.
**Evaluation**	
*friendly*	I felt that the other beings were friendly towards me.
*collaborate*	I felt that it was easy to collaborate with the other beings.
*uncomfortable*	I felt uncomfortable or dizzy in the virtual world.
*likedtask*	I liked the task that I did today in the virtual world.
**Body ownership and agency**	
*mirror*	Although the body that I saw in the mirror did not look like me I had the sensation that it was my body.
*down*	Although the body I saw when I looked down towards myself did not look like me, I had the sensation it was my body.
*mybody*	In general although the body I saw from first person did not look like me I had the sensation that it was my body.
*mymovements*	The body I saw from first person moved in accordance with my movements.

**Fear of death** was assessed through two different questionnaires. The first was a subscale (Death of Self) of the Collett-Lester Fear of Death Scale [40] in its Spanish version [41] (see for example [42] for the use of this subscale in the context of the TMT theory). This consists of 7 questions each on a 1–5 scale where higher numbers indicate greater death anxiety. The overall score is the sum of these 7, therefore with a range of 7 to 35. We refer to this variable as *fod*. The second measure was the Fear of Personal Death Scale [43], see also [13] for the use of this scale in the context of the TMT theory. We translated the items from [43] (p604) into Spanish (note that we adapted item 15, referred to fear of ‘Burial deep in the earth’, to reflect both the option of burial and cremation). This scale consists of 31 questions each on a 1 to 7 scale where higher numbers indicate greater death anxiety. Normally this is subdivided into a number of subscales, but given our limited sample size we decided instead to use the overall average (*fodpersonal*). However, the two scores, *fod* and *fodpersonal* are highly correlated (Spearman’s rho = 0.80, n = 31), and in subsequent analysis it made no difference which of these two were used. Hence in the remainder of this paper we use only *fod*.

**Terror Management Theory** assessment was based on a mortality salience manipulation followed by a measure of worldview defense, in line with previous studies—see [9]. As noted before, prior to the experiment participants were asked about their degree of identification with being Catalan on four questions: (“How important is it to you to be Catalan?”, “To what extent do you see yourself as a typical Catalan person?”, “How well does the term ‘Catalan’ describe you?”, “When you speak of Catalans to with what frequency do you utilise the term ‘We’ rather than ‘They’?”). Each was scored on a 0–10 scale where 10 indicates greater agreement. The overall score (*Catalan*) is the sum of these three (ranging from 0 to 40). This is based on [28] except translated to the context of Catalonia rather than the United States. This variable was used to ensure that those in both the Control and Experimental groups did self-identify with being Catalan.

In session S6 for experimental participants (after the VR session has been completed and participants had virtually died) and on day 1 for control participants, all participants in both groups were induced to think about their own death by requesting them to answer two open questions—e.g., [10, 44]): “Please briefly describe the emotions that the thought of your own death arouses in you” (in Spanish: *Por favor*, *describe brevemente las emociones que te genera el pensar en tu propia muerte*) and “Jot down, as specifically as you can, what you think will happen to you as you physically die and once you are physically dead” (in Spanish: *Por favor*, *explica*, *de la manera más específica posible*, *qué crees que te pasará cuando estés muriendo y una vez estés físicamente muerto/a*) (materials translated from http://www.tmt.missouri.edu/materials.html). This is known as a mortality salience manipulation and it has been widely used in the Terror Management Theory studies [9]. After, participants completed two distracting tasks: the Positive and Negative Affect Scale, PANAS [45]–Spanish translation by [46, 47] and a puzzle, since there is evidence that mortality salience effects are greater after a delay [9]. Finally, participants were asked to read two texts that were presented as if written by immigrants to Catalonia. One of the texts was supposedly written by a person with a positive opinion about Catalonia (*pro*-Catalan text) and the other text by a person with a negative opinion (*anti*-Catalan text) [44], materials were adapted from http://www.tmt.missouri.edu/materials.html, but several changes were introduced in order to fit into the Catalan context, see S2 Text for the texts used). After reading each of the texts (balanced in order) participants were asked to rate, on a scale from 1 to 9, their agreement with 5 statements—e.g. [48, 49]: about the intelligence of the author, about how much they liked the author, about how well informed they thought the author was, about their agreement with the author’s opinion, and about how true they thought the author’s points were (see S2 Text, adapted from www.tmt.missouri.edu/materials.html). The overall score is the sum of the answers for the pro-Catalan text minus the sum of the answers for the anti-Catalan text, thus arriving at a score we refer to as *tmt*, which has a range of -40 to +40.

Another response variable relevant for the TMT hypothesis was an Implicit Association Test (IAT) for racial bias [50, 51]. This is a reaction time test that measures the extent to which participants associate Black or White faces with positive or negative attributes: the faster the associations between Black and Negative and White and Positive compared to Black and Positive and White and Negative, the greater the degree of implicit bias. This is a score (*iat*) in the range -2 to 2 where greater numbers indicate greater bias. This is related to TMT since mortality salience is supposed to lead to greater intergroup bias [52].

**The Life-Changes Inventory** [26] consists of 50 statements (such as “My desire to help others has …”, “My appreciation of nature has …”) and the participant is required to score each one as Strongly Decreased (-2), Decreased Somewhat (-1), Not Changed (0), Increased Somewhat (1), Strongly Increased (2). From these 9 subscales are computed: Appreciation for life (*appreciation*), Self-acceptance (*selfappreciation*), Concern for others (*others*), Concern with worldly achievement (*worldly*), Concern with social/planetary values (*planetary*), Quest for meaning/sense of purpose (*meaning*), Spirituality (*spiritual*), Religiousness (*religious*), Appreciation of death (*dappreciation*). Each of these has the range -2 to 2 being the means of sets of the original scores. There is a final overall score which is referred to as the ‘mean absolute change score’ (*lifechanges*) which is the mean of the absolute values of the 50 individual scores (with therefore the range 0 to 2). It is a measure of the overall amount of change. Whereas in [26] participants have to answer to these statements with the formulation “Since my near-death incident,…” we used the formulation “If I compare my way of seeing life now with the way in which I saw life 8–9 days ago…”. This formulation was used because participants in the control group had not experienced the virtual life-death cycle.

### Statistical methods

The goal was to examine whether there are differences between the Control and Experimental groups on the four response variables: fear of death (*fod*), the Terror Management Theory measure (*tmt*), the Implicit Association Test for racial bias (*iat*), and most important, the life-changes inventory (*lifechanges*). These comparisons are premised on there being a high degree of presence in the virtual environment, and also a high degree of body ownership.

Since we have 4 response variables and also a relatively small sample size, we adopt a Bayesian approach where we can treat all 4 response variables simultaneously in one model. As can be seen in S1 Text the *selfesteem* variable, which had been elicited prior to the VR exposures, differs between the Experimental and Control groups by chance, and thus must be included as a covariate in each equation. This is anyway compatible with the close connection between TMT and self-esteem [9, 12, 53, 54]. The overall model is as follows:

$$\begin{array}{l} fod_{i}\sim N\left(\beta_{fod,0}+\beta_{fod,1}X_{i}+\beta_{fod,2}S_{i},\sigma_{fod}\right)\\ tmt_{i}\sim N\left(\beta_{tmt,0}+\beta_{tmt,1}X_{i}+\beta_{tmt,2}S_{i},\sigma_{tmt}\right)\\ iat_{i}\sim N\left(\beta_{iat,0}+\beta_{iat,1}X_{i}+\beta_{iat,2}S_{i},\sigma_{iat}\right)\\ life\sim N\left(\beta_{life,0}+\beta_{life,1}X_{i}+\beta_{life,2}S_{i},\sigma_{life}\right)\\ i=1,\ldots ,31 \end{array}$$ (1)

*N*(*μ*_*i*_, *σ*) refers to a normal distribution with mean *μ*_*i*_ and standard deviation σ. Here *X*_*i*_ = 1 if the *i*th individual is in the Experimental group and 0 if in the Control group, and *S*_*i*_ denotes the *selfesteem* score.

The prior distributions of the parameters β_*,*j*_, j = 0, 1, …, 3 are Cauchy centred on 0, in order to allow for wide variation. The σ_*_ have improper prior distributions over the range (0, ∞).

The program used for the analysis was Stan (http://mc-stan.org) [55]. The number of chains used was 4, the number of iterations per chain was 4000, and all model fits converged. The results are robust to changes in the likelihood (Eq 1). For example, if the normal distributions are replaced by a Student-t distribution with low degrees of freedom (for example, 5) in order to allow for wider dispersion about the mean, then the results are qualitatively unchanged.

## Results

### Presence, body ownership and evaluations

The questionnaire results on presence, evaluations, and body ownership (Table 1) are applicable only to the Experimental group (n = 15), and their purpose is to check that participants did, overall, experience strong illusions of being on the Island, and that the virtual body was theirs and that the experience was enjoyable.

**Fig 8:**
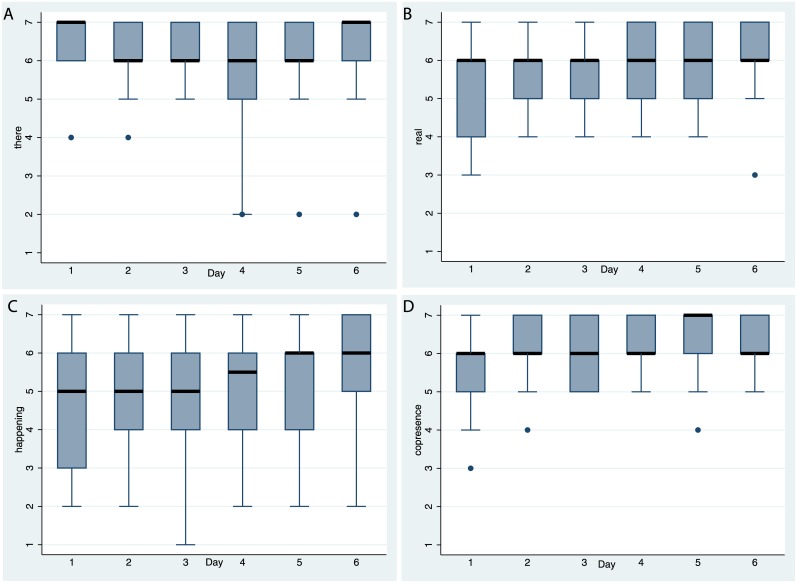
Box plots of the presence questionnaire scores over time. (A) For *there*. (B) For *real*. (C) For *happening*. (D) For *copresence*. (see Table 1). The horizontal thick black lines are the medians, and the boxes the interquartile ranges (IQR). The whiskers extend from max(lowest value, 25^th^ percentile—1.5*IQR) to min(greatest value, 75^th^ percentile + 1.5*IQR). Values outside this range are shown individually.

Fig 8 shows the box plots for the presence questions for each of the 6 exposures. It is clear that the scores on all of the questions are very high, and across all of them over the 6 exposures there is only one where the 25^th^ percentile is lower than 4 (*happening*). For *there*, *real* and *copresence*, the medians are never lower than 6 and for *happening*, never lower than 5. Overall participants had a strong sense of being in the Island environment, that the events were really happening, and with a strong sense of being with the others.

**Fig 9:**
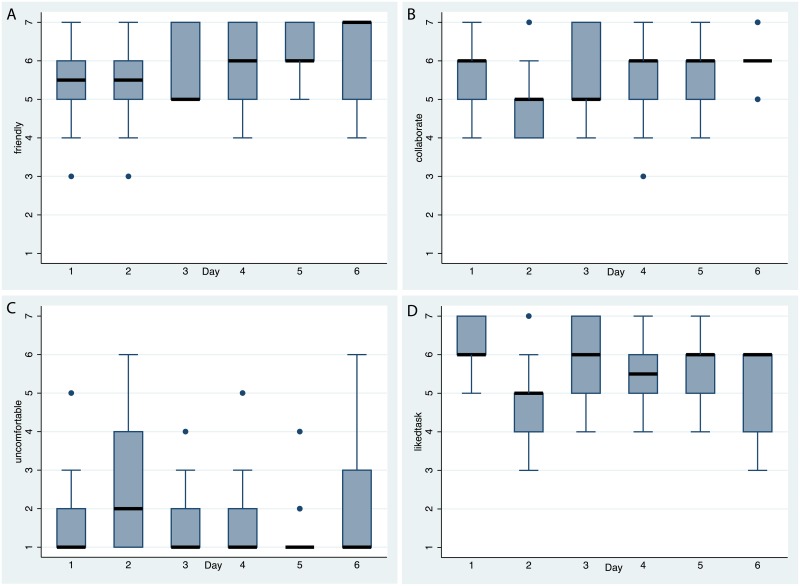
Box plots of the evaluation questionnaire scores over time. (A) For *friendly*. (B) For *collaborate*. (C) For *uncomfortable*. (D) For *likedtask*. See Table 1.

Fig 9 shows that the participants found the others to be friendly, collaborative, and they tended to like the tasks. At the same time there was very little evidence of any systematic discomfort, apart from a few outliers.

**Fig 10:**
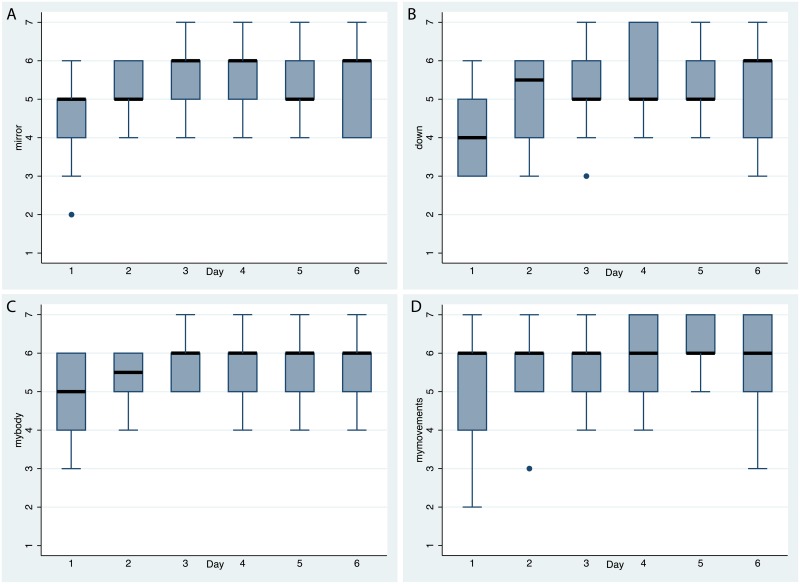
Box plots for body ownership and agency. (A)For *mirror*. (B) For *down*. (C) For *mybody*. (D) For *mymovements*. See Table 1.

Body ownership was high throughout the exposures, in line with previous findings that first person perspective over the virtual body and visuo-motor synchrony provides powerful cues for this illusion. Fig 10 shows the responses, and that even in the first periods (when the participants were with a child-like form) and the last period (when they appeared old and frail) the body ownership scores were high. Fig 10D shows that participants experienced the movements of the virtual body as their own, so that the setup was successful in this regard.

Overall participants experienced the illusions of being in the Island environment, with their virtual body as their own. They liked and collaborated well with the other participants, with very few incidents of discomfort.

### Comparing control and experimental groups on death anxiety, TMT and life changes

Table 2 shows summaries obtained from the posterior distributions of the parameters of the model (Eq 1). It can be seen by examining the β_*,1_ parameters that there is little evidence of any effect of Condition (Control, Experiment) on *iat*, *tmt* and *fod*. However, the posterior probability that the Experimental group had greater *lifechanges* exceeds 0.99. The β_*,2_ parameters show that there is a positive influence of *selfesteem* on both *iat* and *tmt*, but not on *fod* or *lifechanges*. The goodness of fit plots are shown in S3 Text.

**Table 2:** Posterior distributions of the parameters of the model (Eq 1). The first six columns show the means and standard deviations, 2.5^th^, 50^th^, and 97.5^th^ percentiles of the posterior distributions of the parameters of the model. The seventh column shows the posterior probability of the parameter being positive. In the parameters the 0 entry (e.g., *β*_*iat*,0_) refers to the intercept, the 1 entry (e.g., *β*_*iat*,1_**)** is the coefficient of Condition (0 for Control,1 for Experiment) and the 2 entry (e.g. *β*_*iat*,2_) is the coefficient of *selfesteem*.

Parameter	Mean	SD	2.5%tile	Median	97.5%tile	P(β_*_>,0)
**IAT**						
*β*_*iat*,0_	-0.47	0.55	-1.60	-0.45	0.54	0.187
*β*_*iat*,1_	0.11	0.15	-0.20	0.11	0.40	0.766
*β*_*iat*,2_	0.033	0.018	0.000	0.032	0.069	0.976
*σ*_*iat*_	0.41	0.06	0.31	0.40	0.54	
**TMT**						
*β*_*tmt*,0_	-0.23	2.90	-6.77	-0.07	5.10	0.477
*β*_*tmt*,1_	0.53	1.63	-2.29	0.33	4.46	0.618
*β*_*tmt*,2_	0.53	0.10	0.34	0.52	0.75	1.000
*σ*_*tmt*_	8.75	1.18	6.82	8.63	11.42	
**FOD**						
*β*_*fod*,0_	14.85	12.53	-1.37	14.88	38.94	0.888
*β*_*fod*,1_	0.48	1.35	-2.02	0.33	3.67	0.632
*β*_*fod*,2_	0.19	0.39	-0.57	0.19	0.71	0.642
*σ*_*fod*_	6.49	1.01	4.84	6.37	8.79	
**Lifechanges**						
*β*_*life*,0_	0.52	0.36	-0.19	0.52	1.24	0.930
*β*_*life*,1_	0.24	0.10	0.04	0.24	0.42	0.992
*β*_*life*,2_	-0.009	0.012	-0.032	-0.009	0.014	0.219
*σ*_*life*_	0.25	0.03	0.19	0.24	0.32	

To examine *lifechanges* in more detail Table 3 shows the means and standard errors of the subscales, ordered in increasing magnitude of the (absolute value) of Cohen’s d. The subscales *meaning*, *selfacceptance*, and *others* show the clearest differences, with those in the Experimental group having greater scores than those in the Control group.

**Table 3:** Means, standard errors and Cohen’s d comparing the life changes subscale Items between control and experimental groups.

	Control		Experimental		
Variables (subscales)	mean	SE	mean	SE	Cohen’s d
*appreciation*	0.23	0.077	0.30	0.098	-0.19
*worldly*	0.02	0.062	-0.04	0.070	0.22
*planetary*	0.21	0.069	0.28	0.089	-0.22
*dappreciation*	0.04	0.041	0.11	0.090	-0.26
*spiritual*	0.13	0.044	0.03	0.107	0.31
*religious*	0.00	0.023	-0.22	0.172	0.46
*meaning*	0.30	0.089	0.58	0.154	-0.59
*selfacceptance*	0.31	0.107	0.62	0.155	-0.60
*others*	0.24	0.085	0.46	0.092	-0.64
**(overall)**					
*lifechanges*	0.25	0.046	0.47	0.071	-0.91

### The influence of body ownership and death anxiety

One premise of this research is that a high level of body ownership over the several sessions can enhance the effectiveness of the scenario as it relates to personal death. It is implicitly addressing the point: ‘This was my body, I died in that body, I experienced the NDE, but I am still alive in this (physical) world’. Therefore it is interesting to consider the impact of body ownership on the four response variables. In order to create one overall score we averaged all the scores over the three variables *mirror*, *down*, *mybody* over the last 3 sessions (the result is almost identical to extracting the first component from a factor analysis). We refer to this as *meanownership*. In addition according to TMT the critical variable should be death anxiety (*fod*). Therefore, we examine a new model, restricted to the experimental group only, where we consider the influence of both *meanownership* and *fod*.

In the model shown in Eq 2, *O* refers to *meanownership* and *F* to *fod*. We use the same prior distributions as for Eq 1.

$$\begin{array}{l} F_{i}=fod_{i}\sim N\left(\beta_{fod,0}+\beta_{fod,1}O_{i},\sigma_{fod}\right)\\ tmt_{i}\sim N\left(\beta_{tmt,0}+\beta_{tmt,1}O_{i}+\beta_{tmt,2}F_{i},\sigma_{tmt}\right)\\ iat_{i}\sim N\left(\beta_{iat,0}+\beta_{iat,1}O_{i}+\beta_{iat,2}F_{i},\sigma_{iat}\right)\\ life\sim N\left(\beta_{life,0}+\beta_{life,1}O_{i}+\beta_{life,2}F_{i},\sigma_{life}\right)\\ i=1,\ldots ,15 \end{array}$$ (2)

From the FOD block we can see the *fod* is not related to *meanownership*. The *iat* is negatively associated with *meanownership* (the posterior probability that the coefficient is negative is 0.93) but not associated with *fod*. The *tmt* is positively associated with *fod*, but there is not very strong evidence that it is related to *meanownership*. Finally there is strong evidence that *lifechanges* is positively related both to *meanownership* and *fod*. Goodness of fit results are shown in S3 Text.

Overall these goodness of fit tests show that the models above do not explain at all the variation in *fod*, being unrelated to Condition and *selfesteem* (Eq 1) and unrelated to body ownership (Eq 2).

### Evaluation of the experience

Although the Island experience was designed to be a positive one, involving participants carrying out tasks with compatriots in a beautiful island setting, it may be thought that the portrayal of the deaths of their compatriots, and finally their own personal death, would lead to unpleasant feelings. After their final session (S6) participants were asked: “What thoughts, sensations or emotions have you experienced while you were in the virtual environment?” as part of a general interview. The results of those interviews are the subject of a further paper, but a content analysis of typical words used in answer to this specific question included the occurrence of the word ‘sadness’ 7 times amongst the 14 participants who responded, ‘death’ 10/14 times, ‘good/liked’ 7/14 with the remaining lower frequency words being ‘afraid/scared’ (3), ‘curiosity’ (2), and ‘rare’ (2).

Fifteen days after their final exposure participants were sent an email that had three questions with the results shown in S1 Table, based on the 17 responses received, almost all positive.

These reports suggest that participants on the whole did not have unpleasant or distressful experiences.

## Discussion

This paper provides a methodology that allows ‘death’ to be treated as an independent variable, and thus supports controlled experiments in areas such as Terror Management Theory, and also for examining the consequences of events such as NDEs—that cannot be accomplished in reality. The work relies on the power of VR to deliver three fundamental illusions: Place Illusion (the illusion of being in the virtual place), Plausibility (the illusion that the events are really occurring) [17] and Body Ownership (the illusion of ownership over the virtual body that is seen from first person perspective as visually substituting the person’s own body) [56–58]. Unlike almost all other research in the area of presence (Place Illusion and Plausibility) and body ownership in VR, participants were exposed multiple times, and the results showed that these illusions were strongly experienced throughout. Place Illusion has been argued to rest on the foundation of natural sensorimotor contingencies for perception [17]. In this case participants perceived through a stereo wide field-of-view head-mounted display (albeit with relatively low resolution) with 6 degrees of freedom head-tracking. The events on the Island and the narrative were sufficient to maintain a high degree of Plausibility (questionnaire variables *real* and *happening*)–in particular Plausibility has been argued to rest on the environment responding to the actions of the participant, events that directly and personally relate to the participant—both of which were strongly represented in the Island scenario. The third requirement of Plausibility is that when the environment depicts something that occurs in reality then it should conform to expectations. However, this issue was not relevant, since the environment was a fantastic one, deliberately made other-worldly to emphasise that it was on a different ‘plane’ to reality. Nevertheless all the elements of the scenario were internally consistent and coherent, which has been shown to be another requirement for Plausibility [59].

Body ownership was high throughout, in spite of the virtual body being humanoid but not human, and at various different ages. This supports other findings that adults can have a strong illusion over a virtual child body [39] or even a Barbie doll [60], a different raced body [25, 61] including a purple coloured body [23]. The first person perspective [62] and multisensory integration [58], and especially visuomotor synchrony between body movements and movements of the virtual body [63] all contribute to the illusion of body ownership. What is especially new here is that we showed that the illusion was sustained over multiple exposures.

Additionally, unlike other work in this area, participants were in a virtual environment that they shared with two other people, carrying out tasks together, where they repeatedly encountered the same people, whom they saw grow old and die, before they themselves experienced ‘dying’ out of the environment. The level of co-presence, the illusion of being with others, was high throughout, and the others were experienced as being likeable, and doing enjoyable tasks together.

All of the above elements were essential to our main goal of creating a fantastic strange alternate reality that people would come to take as an alternative life, that they would then experience leaving once and for all, after having the simulated death experience. The results show that these elements were realised successfully.

Now we consider each of the response variables—death anxiety, IAT, TMT, and life-changes in turn. The measure of death anxiety (*fod*) was not influenced by Condition (Control, Experiment), *selfesteem* (Table 2) nor the level of body ownership (Table 4). In [27] we found that a type of virtual out-of-body experience did result in a reduction of fear of death. That was a single issue study where there was just one factor manipulated—the precise method by which the out-of-body experience was generated. The present study has multiple elements—seeing the death of others, the out-of-body experience, the simulated NDE and all the social interactions and events that had happened beforehand. Also in [27] the fear of death was assessed straight after the VR experience whereas in this case one day later. Therefore it is not too surprising that the same result was not obtained.

**Table 4:** Posterior distributions of the parameters of the model (Eq 2). The first six columns show the means and standard deviations, 5^th^, 50^th^, and 95^th^ percentiles of the posterior distributions of the parameters of the model. The seventh column shows the posterior probability of the parameter being positive.

Parameter	Mean	SD	5%	median	95%	P(β_*_>,0)
**IAT**						
*β*_*iat*,0_	1.63	0.89	-0.11	1.64	3.37	0.967
*β*_*iat*,1_	-0.17	0.11	-0.39	-0.17	0.06	0.070
*β*_*iat*,2_	0.001	0.020	-0.037	0.001	0.042	0.518
*σ*_*iat*_	0.31	0.08	0.20	0.30	0.49	
**TMT**						
*β*_*tmt*,0_	0.61	3.86	-5.34	0.15	11.31	0.546
*β*_*tmt*,1_	1.22	1.38	-0.98	1.01	4.25	0.825
*β*_*tmt*,2_	0.51	0.35	-0.23	0.54	1.14	0.917
*σ*_*tmt*_	10.88	2.40	7.37	10.51	16.53	
**FOD**						
*β*_*fod*,0_	21.48	8.75	-0.01	22.65	36.13	0.975
*β*_*fod*,1_	0.08	1.55	-2.57	-0.12	3.93	0.448
*σ*_*fod*_	5.06	1.23	3.32	4.84	8.14	
**Lifechanges**						
*β*_*life*,0_	-1.27	0.62	-2.44	-1.28	-0.02	0.023
*β*_*life*,1_	0.14	0.08	-0.02	0.15	0.30	0.959
*β*_*life*,2_	0.043	0.014	0.013	0.043	0.071	0.995
*σ*_*life*_	0.22	0.05	0.14	0.21	0.34	

The IAT for racial bias against Black people (*iat*) was positively associated with *selfesteem* (Table 2). The *iat*, however, was negatively associated with body ownership (Table 4). Peck, Seinfeld, et al. [23] found that although White participants experienced body ownership over a purple body this did not influence the IAT for racial bias, whereas their embodiment in a Black body did result in a reduction of bias as measured by the IAT. However, in the case of the Island the virtual bodies were not human bodies with another colour but humanoid, quite clearly of a different ‘species’. Moreover, there was positive contact throughout with the other characters who were also of the same species, though different colours. We speculate that the reduction in bias associated with the stronger illusion of body ownership may have been caused by a combination of this ‘other species’ embodiment, and the contact hypothesis, which argues that positive contact with others of a different race maps over to a general decline in racial bias [64, 65].

The TMT also showed no effect of Condition, but did increase with *selfesteem* (Table 2). The relationship between TMT and self-esteem is a complex one. When self-esteem is manipulated within an experiment, i.e., through a bogus personality test that reports positive attributes to some people and negative to others, then the evidence suggests that it mitigates against mortality salience effects [54]. Similarly if implicit measures of self-esteem are used (e.g., asking people to rate the attractiveness of letters and measuring the extent to which preference is given to their own initials) then again such measured high self-esteem acts against mortality salience effects [53]. The argument is that such self-esteem acts as an anxiety buffer thereby modulating the effects of mortality salience. However, as noted in [9] those studies that used the self-report Rosenberg scale to measure self-esteem find the opposite effect, that higher self-esteem is associated with an enhancement of the mortality salience effect. They point out that “One explanation for these differing self-esteem findings is that self-reported versus other methods for assessing self-esteem may measure different facets of the construct, and explicit or self-reported self-esteem may not buffer the effects of mortality as TMT has posited”. Moreover, high self-reported esteem may not really measure just self-esteem but rather express other factors including narcissism and insecurity, for which Burke, Martens, et al. [9] present evidence.

Also supporting the idea of Terror Management Theory is the positive association between *tmt* and *fod* (Table 4): in other words the more people were inclined to support their nation the greater their fear of death subsequent to the experiment.

The life changes inventory (*lifechanges*) tended to be higher for those in the Experimental compared to the Control group (Table 2). Table 3 suggests that the most important elements of this were the subscales *meaning* (quest for meaning/sense of purpose), self-acceptance and concern for others. Moreover, Table 4 shows that the *lifechanges* score was positively associated with both body ownership and fear of death.

It was highly interesting to note that participants were never explicitly trained to carry out the tasks or the ceremony at the death of one of the partners. This knowledge was introduced by the first confederates acting as participants in the early phases of the experiment, and then handed down the generations, so that by the time there were no confederates remaining the participants were managing themselves with the older ones guiding the younger ones. Anecdotally, when the participants finally met together days after the experiment in an overall debriefing meeting (S8), this was a quite emotional reunion. Recall that they had never met physically, and knew nothing about one another, not even that they were all women. Participants exchanged stories of their time in the Island and shared their experiences and feelings with one another and the experimental facilitator. This, together with the results of their post experimental interviews, and reports from the daily diary kept by participants, will be the subject of a further report.

As mentioned in Section 4.4, and as we found as a result of the debriefing meeting, participants overall had positive experiences. Nevertheless, there are important ethical issues in using VR for experimental studies, as discussed by Madary and Metzinger [66]. In the current study the environment and most experiences were designed to be pleasant, participants were able to leave at any time without giving reasons, they gave informed consent, and vulnerable people were excluded. Brey [67] reviewed ethical issues associated with virtual reality and argued that it is a fundamental moral principle “that human beings have a duty to treat other persons with respect, that is, to treat them as ends and not as means, or to do to them as one would expect to be treated by others oneself.” He then went on to argue that in VR this applies also to virtual characters. In our experiment participants were actually shown how to construct a monument to remember their partners who had died, so that they were indeed respected.

We have seen in this and other studies that VR may lead to changes in implicit attitudes and behaviours in participants. Up to now, to our knowledge, this has only been for what most would consider as positive reasons—e.g. diminishing racial bias [24], or the aim of reducing domestic violence [68] as two instances. In this experiment such changes were concerned with attitudes towards self-acceptance or caring for others, again positive changes. Unfortunately, the same methodology could be used for malfeasance (and this applies to any technology). Researchers have the duty to be aware of and to guard against such a possibility.

Although our research is based on the premise that NDEs are generally associated with positive life changes, it should be noted that this is not always the case. For example, OBEs may involve seeing a double of oneself (he-autoscopy) which can be particularly disorienting and terrifying—see the case report in [69]. There are some reports of fear or distress during OBEs and NDEs [70, 71]. A small number of distressing experiences were reported by burns survivors who experienced NDEs, but most do report positive NDEs [37].

Given the complexity of this experiment, both with respect to producing the software involved, the logistics of the study, the resources available, and that this was the first time that anything like this has ever been done, the sample size was inevitably small. In the Bayesian analysis we have used high variance prior distributions (in fact the Cauchy distribution used for the parameters has infinite variance) and yet we nevertheless found posterior probabilities indicating possible relationships.

It is important, nevertheless, to be aware of the limitations of this study. The experiment needs to be repeated with a greater sample size, and also should include men as well as women, and various ages. The experiment was particularly complex, and our goal was to demonstrate the fundamental idea and methodology and to show that this was feasible. Hence, we only included women since this removed one element of possible variation that might have been introduced were both sexes to have been included. The important point of this paper is that we have shown that it is possible to utilise virtual reality for studies involving death, even personal death, and we hope that this has opened up an exciting new field in which others can follow.

The approach that we have adopted has wider implications. Death might be considered the most extreme example of a “transformative experience” [72, 73]. For example, when making a critical life decision, such as whether or not to have a child, we cannot know how that would be without actually having the experience. We typically imaginatively project ourselves forward into possible futures, and try to evaluate the possible outcomes of different decisions. However, this is especially complex since the experience itself may fundamentally change us, so that the way we evaluate the results of that decision while living its consequences may be quite different from any prior evaluation based on imaginal future projections. Our experiment points to the possibility for people to gain insight into such transformative experiences through simulation of alternate futures. The technology is not available yet to offer personalised experiences (constructing such environments today remains significant team work over extended periods of time), but a system for personalised VR experiences useful for decision making is a capability worth pursuing.

## Supporting information

S1 Text The sample—A summary of the demographic variables. (DOCX)

S2 Text Pro and anti Catalan texts used, together with the evaluation questions for both texts (Spanish). (DOCX)

S3 Text Goodness of fit plots. (DOCX)

S1 Table Responses to email sent 15 days after the final day of the experiment. (DOCX)

S1 Data The data set. Some individual defining characteristics such as age and religion have been removed for data protection reasons.
(CSV)
