# Supplementary material for: Virtual mortality and near-death experience after a prolonged exposure in a shared virtual reality may lead to positive life-attitude changes
Source: PLoS One. 2018 Nov 5;13(11):e0203358. doi: 10.1371/journal.pone.0203358 (PMC6218023; doi:10.1371/journal.pone.0203358)
Supplement: S1 Text — (DOCX) [file pone.0203358.s001.docx]

The Island - A Life and Death Experience in a Shared Virtual Reality

Itxaso Barberia, Ramon Oliva, Pierre Bourdin, Mel Slater

# S1 Text – The Sample

There were 31 female participants, all undergraduate students. There were 16 in the Control group and 15 in the Experimental Group. 10 in each group had corrected vision, and the remainder good vision without correction.

**Table A** – Characteristics of the Sample shown as median (Interquartile Range) for ordinal variables and mean ± SD for numerical variables.

| **Variable** | **Meaning** | **Control** | **Experiment** |
| --- | --- | --- | --- |
| IT | Level of knowledge of IT on a 1(low) to 7 (high) scale | 4 (1) | 4 (2) |
| Programming | Level of knowledge of computer programming on a 1(low) to 7 (high) scale. | 1 (1.5) | 2 (2) |
| Gamesyear | See below | 2 (1.5) | 2 (2) |
| Gamesweek | See below | 1.5 (1) | 2 (1) |
| Age |  | 20 ± 1.5 | 20 ± 1.4 |
| Catalan^1^ | (Huddy and Khatib, 2007) scale in relation to Catalonia. | 27.8 ± 5.1 | 30.2 ± 5.3 |
| Selfesteem^2^ | Rosenberg Self Esteem scale (Rosenberg, 1989) | 30.9 ± 3.6 | 33.6 ± 3.9 |

| Gamesyear (number of times)  1: Never  2: 1-5  3: 6-10  4: 11-15  5: 16-20  6: 21-25  7: 25+ | Games week (hours played)  1: 0  2: <1  3: 1-3  4: 3-5  5: 5-7  6: 7-9  7: 9+ |
| --- | --- |

^1^ Cohen’s d = 0.46 for the difference in Catalan between Control and Experimental groups.

^2^ Cohen’s d = 0.71 for the difference in selfesteem between Control and Experimental groups.

**Table B** – Participant Religious Belief

| **Religious belief** | **Control** | **Experiment** | **Total** |
| --- | --- | --- | --- |
|  |  |  |  |
| Atheist | 10 | 8 | 18 |
| Agnostic | 4 | 3 | 7 |
| Non-practicing believer | 0 | 3 | 3 |
| Practicing believer | 1 | 1 | 2 |
| Other | 1 | 0 | 1 |
|  |  |  |  |
| Total | 16 | 15 | 31 |
