# Supplementary material for: Virtual mortality and near-death experience after a prolonged exposure in a shared virtual reality may lead to positive life-attitude changes
Source: PLoS One. 2018 Nov 5;13(11):e0203358. doi: 10.1371/journal.pone.0203358 (PMC6218023; doi:10.1371/journal.pone.0203358)
Supplement: S2 Text — (DOCX) [file pone.0203358.s002.docx]

The Island - A Life and Death Experience in a Shared Virtual Reality

Itxaso Barberia, Ramon Oliva, Pierre Bourdin, Mel Slater

# S2 Text – Pro and anti Catalan texts used, together with the evaluation questions for both texts

Pro Catalan text:

Lo primero que me llamó la atención cuando llegué a Cataluña fue la gran hospitalidad y simpatía de la gente. La gente aquí te respeta mucho y aunque vengas de una cultura diferente te escuchan y siempre intentan entenderte. De donde yo vengo las cosas no son tan fáciles. Creo que los catalanes son una sociedad muy amable y trabajadora. Te valoran por el esfuerzo que haces y no por cuál es tu lugar de origen, tienen un sentido de la justicia muy arraigado. En general creo que la sociedad catalana es admirable y no me arrepiento de haber venido.

Anti Catalan text:

Al principio, cuando llegué a Cataluña, pensaba que éste era un lugar lleno de oportunidades, pero pronto me di cuenta de que esto es verdad sólo para la gente de aquí. El sistema está montado para favorecer a los de aquí y dificultar las cosas a los que venimos de fuera. De lo único que se preocupan los catalanes es de su dinero y de tratar de tener más dinero que otras personas. No piensan en los demás. La gente es muy fría. Nadie nos tiene en cuenta.. La gente de aquí son niños mimados y vagos y esperan que las cosas les caigan del cielo. Cataluña es un lugar frío e insensible a las necesidades y problemas de los que venimos de fuera. Creía que era un lugar genial pero me equivoqué.

Evaluation questions:

Responde a las siguientes preguntas indicando en una escala del 1 al 9 hasta qué punto te parece que el párrafo anterior más encaja con tu opinión.

-¿Hasta qué punto te gusta la persona que ha escrito este texto?

Un valor de 1 significa “Nada” y un valor de 9 significa “Mucho”.

-¿Hasta qué punto piensas que se trata de una persona inteligente?

Un valor de 1 significa “Nada inteligente” y un valor de 9 significa “Muy inteligente”.

-¿Hasta qué punto crees que esta persona está bien informada?

Un valor de 1 significa “Muy mal informada” y un valor de 9 significa “Muy bien informada”.

-¿Hasta qué punto estás de acuerdo con la opinión de esta persona?

Un valor de 1 significa “Nada de acuerdo” y un valor de 9 significa “Muy de acuerdo”.

-¿Hasta qué punto consideras que la opinión de esta persona encaja con la realidad?

Un valor de 1 significa “Nada” y un valor de 9 significa “Mucho”.
