# Supplementary material for: Virtual mortality and near-death experience after a prolonged exposure in a shared virtual reality may lead to positive life-attitude changes
Source: PLoS One. 2018 Nov 5;13(11):e0203358. doi: 10.1371/journal.pone.0203358 (PMC6218023; doi:10.1371/journal.pone.0203358)
Supplement: S3 Text — (DOCX) [file pone.0203358.s003.docx]

The Island - A Life and Death Experience in a Shared Virtual Reality

Itxaso Barberia, Ramon Oliva, Pierre Bourdin, Mel Slater

# S3 Text – Goodness of Fit

## The model based on EQN 1

The Stan library was used to generate new data in conformance with the estimated model. An abstract representation of the code is as follows:

for *i* = 1 to *n* {

$${fod'}_{i}= Nrng({\beta'}_{fod,0}+{\beta'}_{fod,1}X_{i}+{\beta'}_{fod,2}S_{i}, {\sigma'}_{fod})$$

$${tmt'}_{i}= Nrng\left( {\beta'}_{tmt,0}+{\beta'}_{tmt,1}X_{i}+{\beta'}_{tmt,2}S_{i}, {\sigma'}_{tmt} \right)$$

$${iat'}_{i}= Nrng\left( {\beta'}_{iat,0}+{\beta'}_{iat,1}X_{i}+{\beta'}_{iat,2}S_{i}, {\sigma'}_{iat} \right)$$

$$life^{'}= Nrng\left( {\beta'}_{life,0}+{\beta'}_{life,1}X_{i}+{\beta'}_{life,2}S_{i}, \sigma_{'life} \right)$$

}

where *n* = 31 is the number of participants.

The $'$ marked variables on the left hand side indicate that these new generated variables are delivered by a normal random number generator *Nrng* with the given means and standard deviations. The $'$ on the right hand side indicates that the corresponding parameters are replaced by their posterior distributions. In other words, for each ${fod'}_{i}$ (for example) we obtain a simulated probability distribution, and the simulation size is N = 8000. The same is true for the other variables.

In order to check goodness of fit we can find the means (of the samples of 8000) for each ${fod'}_{i}$ and plot these against the corresponding observed ${fod}_{i}$ (and do the same for each of the other variables). The size of the correlation coefficient acts as an effect-size. Below we plot each scatter diagram, and give the corresponding correlation coefficient.


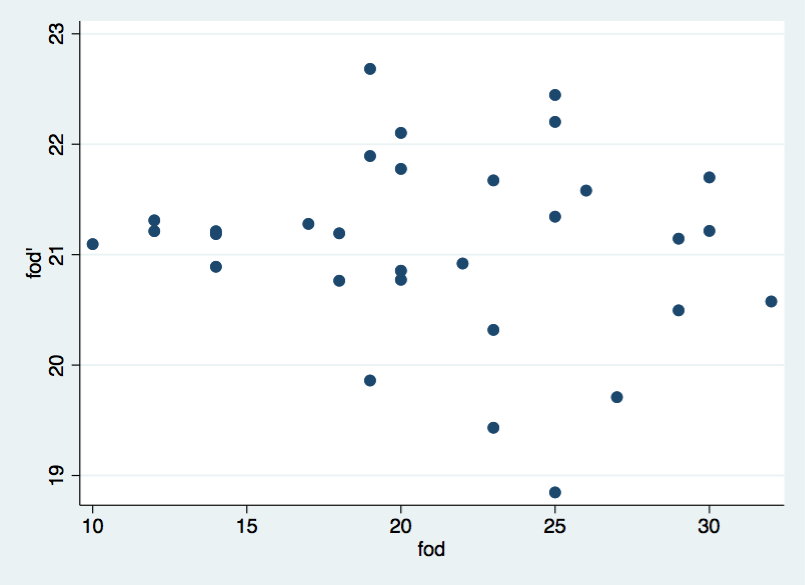


Figure A – Predicted fod$'$ by observed fod. r = -0.12, n = 31.


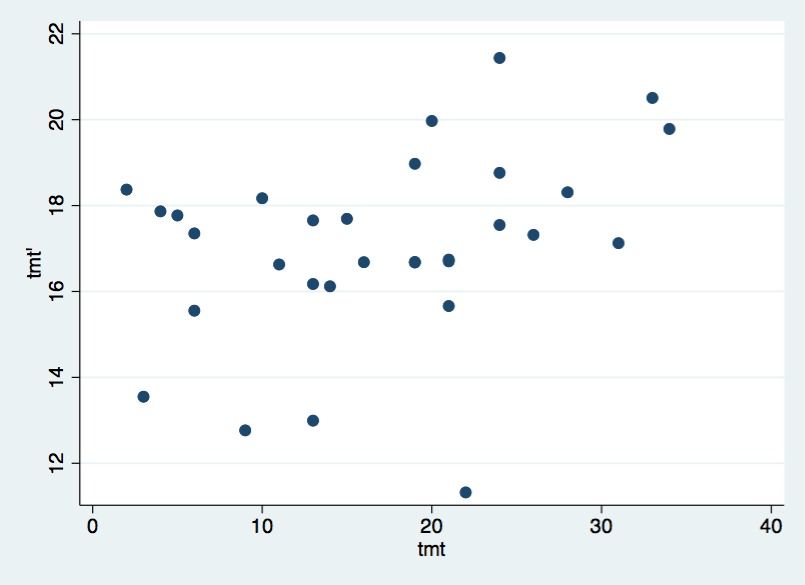


Figure B – Predicted tmt$'$ by observed tmt. r = 0.36, n = 31.


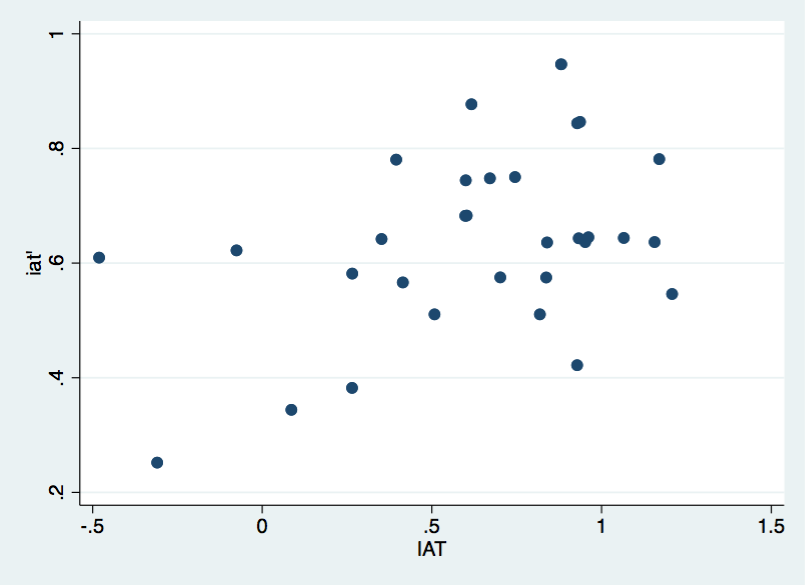


Figure C – Predicted iat$'$ by observed iat. r = 0.41, n = 31.


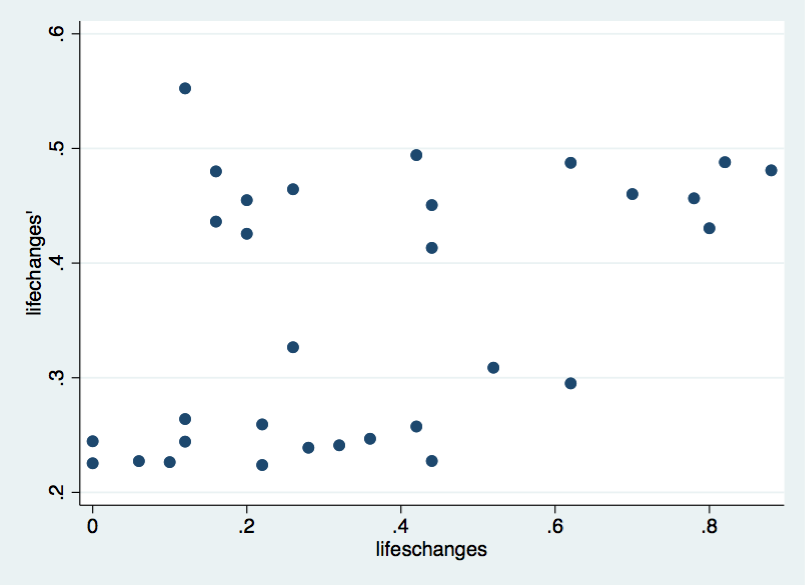


Figure D – Predicted lifechanges$'$ by observed lifechanges. r = 0.45, n = 31.

Apart from *fod*, which has a small effect size, the effect sizes are in the range medium (0.30) to large (0.50). We conclude that *fod* is not influenced by the model, neither by the Condition nor *selfesteem*, which is also evident in Table 2.

## The model based on EQN 2

These data are restricted to the Experimental group only (n = 15), otherwise the same method as above is used.


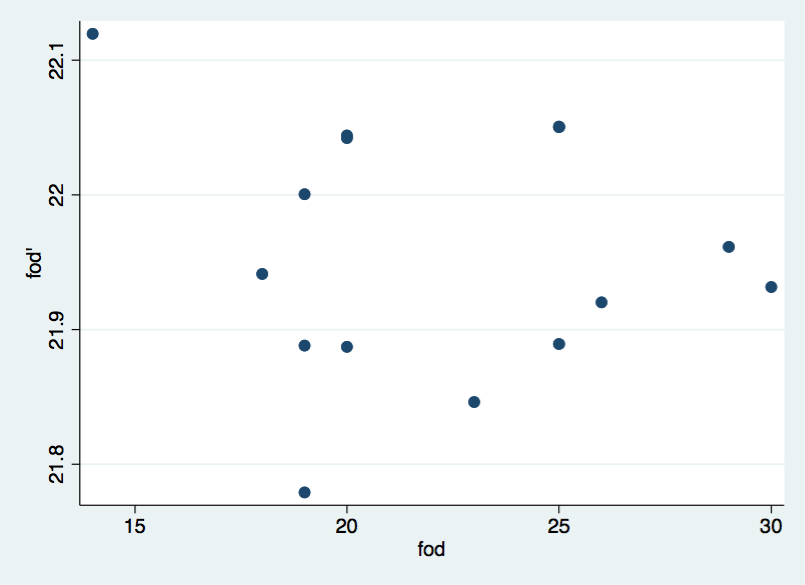


Figure E – Predicted fod$'$ by observed fod. r = -0.16, n = 15.


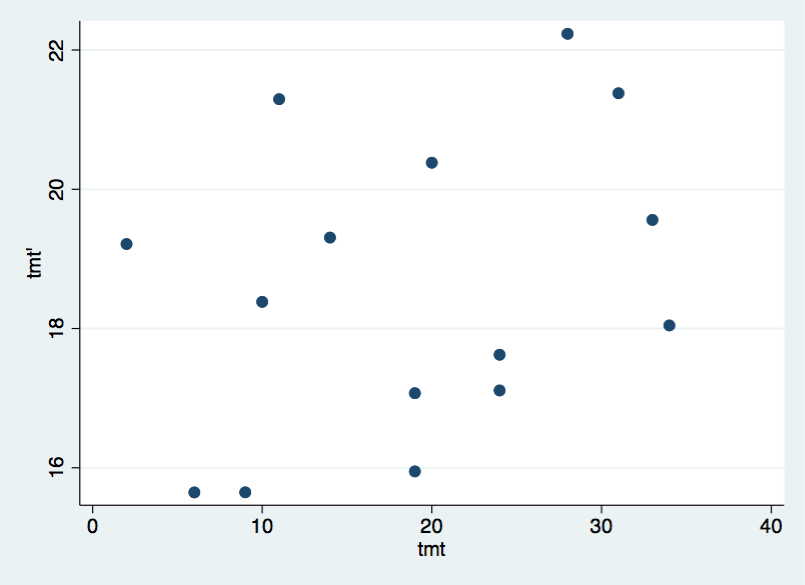


Figure F – Predicted tmt$'$ by observed tmt. r = 0.32, n = 15.


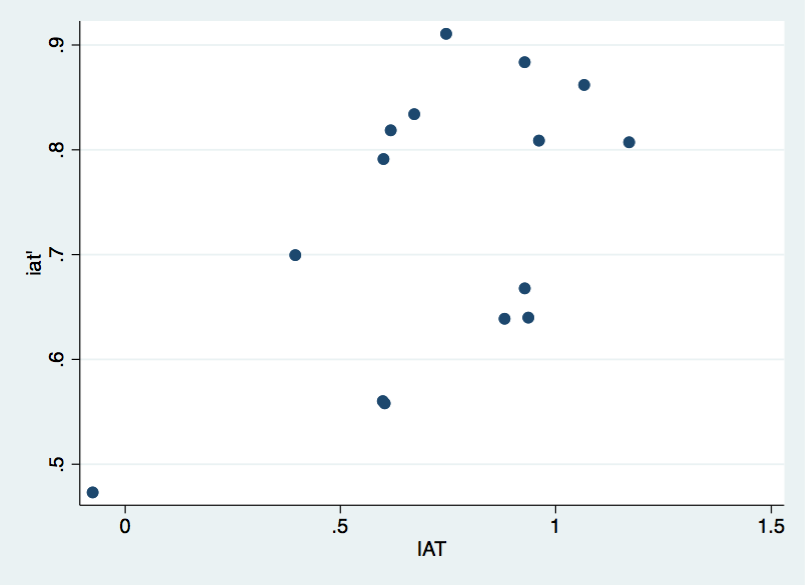


Figure G – Predicted iat$'$ by observed iat. r = 0.55, n = 15.


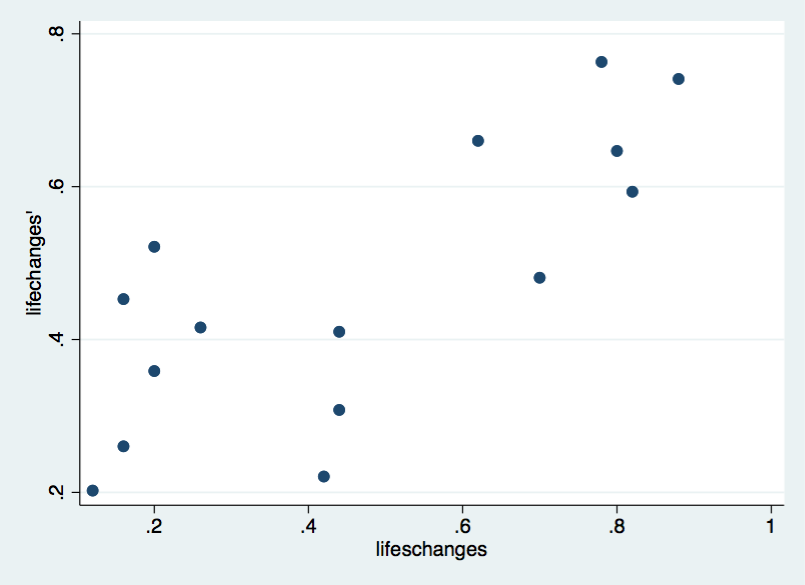


Figure H – Predicted lifechanges$'$ by observed lifechanges. r = 0.77, n = 15.

As before the *fod* variable is not well predicted by the model with a small effect size. The effect size for *tmt* is medium, and large for both *iat* and *lifechanges*.
