# Supplementary material for: Virtual mortality and near-death experience after a prolonged exposure in a shared virtual reality may lead to positive life-attitude changes
Source: PLoS One. 2018 Nov 5;13(11):e0203358. doi: 10.1371/journal.pone.0203358 (PMC6218023; doi:10.1371/journal.pone.0203358)
Supplement: S1 Table — (DOCX) [file pone.0203358.s004.docx]

The Island - A Life and Death Experience in a Shared Virtual Reality

Itxaso Barberia, Ramon Oliva, Pierre Bourdin, Mel Slater

**S1 Table** – Responses to Email 15 days after the final day of the experiment. 31 emails were sent out and 17 responses received, 7 in the Control Group and 10 in the Experimental Group. Not all responding participants answered all questions.

|  | Control Group | | Experimental Group | |
| --- | --- | --- | --- | --- |
| Question | No | Yes | No | Yes |
| “Have you had any thoughts or negative reactions regarding the experiment?” | 4 | 1 | 10 | 0 |
| Have you had any positive thoughts or reactions regarding the experiment? | 2 | 1 | 1 | 9 |
| In the future, would you like to participate in a similar study again? | 0 | 7 | 0 | 10 |
